# α‑Ketol Rearrangement for Accessing Tetracyclic Natural Products

**DOI:** 10.1021/acs.orglett.5c02235

**Published:** 2025-07-22

**Authors:** Alexandru Sara, Ulrike Eggert, Markus Kalesse

**Affiliations:** † Institute of Organic Chemistry, 26555 Gottfried Wilhelm Leibniz Universität Hannover, 30167 Hannover, Germany; ‡ Centre of Biomolecular Drug Research (BMWZ), Wilhelm Leibniz Universität Hannover, 30167 Hannover, Germany

## Abstract

Tetracyclic natural products, which utilize the tetracycline skeleton, demonstrate a diverse array of biological activities. A critical structural feature of these compounds is the tertiary alcohol situated within a *cis*-decalin framework. In this study, we present a comprehensive protocol for the synthesis of tricyclic building blocks and complete carbon frameworks. This methodology facilitates the stereoselective synthesis of a specific enantiomer as well as its inversion to the corresponding opposite isomer via a ketol rearrangement.

## Graphical abstract

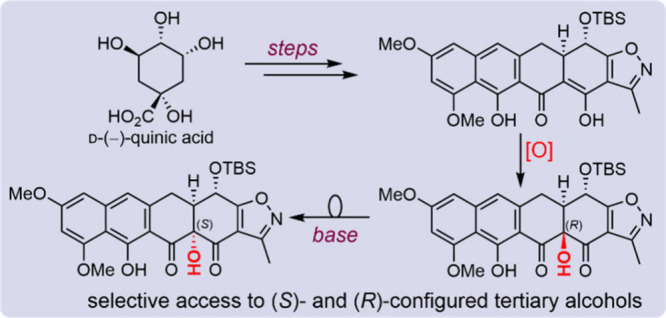

A wide array of biologically active natural products contain the fundamental framework of tetracyclines.^1,2^ Of these, the tetracycline family has become one of the most widely prescribed classes of antimicrobial agents.^3,4^ In the context of combating antimicrobial resistance (AMR), in 2015, chelocardin (**1**) (Scheme 1a), described by the research groups of Müller and Petkovic, was identified as a notable variation of the tetracycline skeleton due to its distinctive amino group configuration.^5,6^ The natural product has initially garnered interest as a promising lead compound due to its remarkable antibacterial activity against pathogens of the ESKAPE panel, with its framework-sharing parallels with mithramycin, a U.S. Food and Drug Administration (FDA)-approved aureolic-acid-derived RNA synthesis inhibitor utilized in the treatment of testicular cancer.^7–9^ While the class of aureolic acids, bearing a characteristic annulated 6/6/6-tricyclic framework, lacks the fourth cycle present in the tetracycline naphthacene core, the structural relationship between these two classes of polyketide natural products becomes more evident when analyzing their biosynthetic paths.

**Scheme 1:**
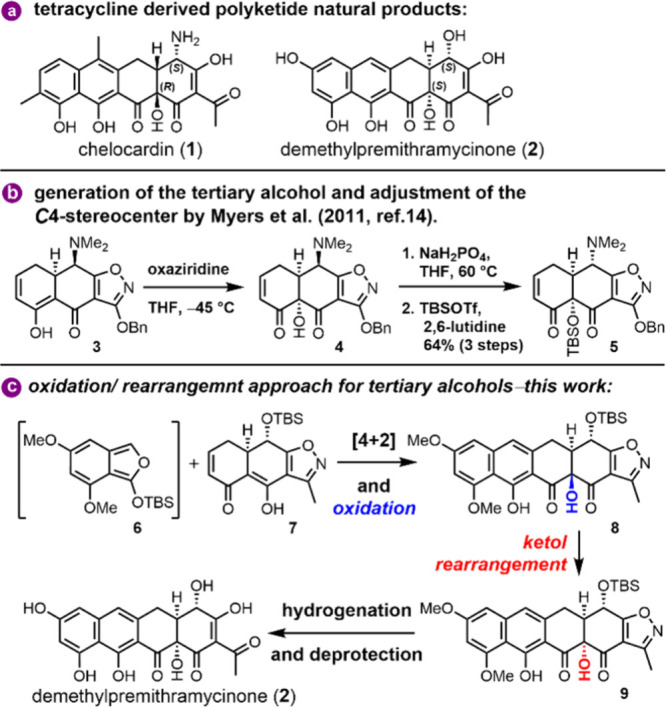
(a) Tetracyclic Polyketide Natural Products of Bacterial Origin, (b) Myers Approach for C8a Functionalization and *C*4 Adjustment, and (c) C12a-Alcohol Generation with Subsequent Inversion of the Configuration

While the isolation of chromomycin A_3_ by Miyamoto et al., as the first member of the aureolic acid family, was achieved already in 1964,^10^ with further derivatives emerging in the following years, a breakthrough in terms of biosynthesis was achieved in 1998 by Rohr et al. With their isolation and characterization of demethylpremithramycinone (**2**), a tetracyclic intermediate of the biosynthetic pathway, the biosynthetic relationship between aureolic acids and the tetracycline family was established.^11^

Inspired by previous total syntheses of tetracyclines as reported by Tatsuta et al.,^12^ Myers and co-workers,^13,14^ and Stork et al.,^15^ the stereoselective, convergent strategy for accessing the natural product demethylpremithramycinone (**2**) relied on both established protocols as well as original approaches for overcoming long-known challenges. While the majority of the published tetracycline syntheses of the past two decades mainly rely on Michael–Dieckman cyclizations for accessing the characteristic naphthacene skeleton,^12,13,15^ a Diels–Alder approach with isobenzofuran was envisioned here as a key transformation (Scheme 1c). By using the strategy implemented by Stork and Hagedorn in 1978 of utilizing isoxazoles as masking agents for 1,3-diketone functionalities,^16^ the final decorations of the A ring arise from the hydrogenolytic cleavage of the isoxazole motif of **9**. Further, specific removals of additional protective groups, such as silyl and methyl ethers, required for the protection of alcohol and phenol functionalities are also to be cleaved during the final stages. Envisioning a late-stage derivatization of the C12a position, the tertiary alcohol can be traced back to a direct Rubottom oxidation. With bulky peroxides employed, this transformation is expected to yield the undesired (*R*)-configured tertiary alcohol due to steric hindrance exerted by the C4 functionality. In order to overcome this impediment, an α-ketol-type rearrangement was envisioned for correcting the configuration of the C12a-alcohol. Nevertheless, the ability to generate both (*R*)- and (*S*)-configured tertiary alcohols at C12a represents an additional noteworthy asset of the envisioned strategy in the context of accessing new tetracycline-derived compounds with bioactive potential.

Further on, the aromatic C ring arises from an elimination, performed subsequently to the naphthacene core construction.

Finally, the advanced tetracycline precursors can be traced back to a [4 + 2]-cycloaddition reaction between decalin-enone **7**, completed on a multigram scale from d-(−)-quinic acid over 11 linear steps with an overall yield of 28%, and *in situ* generated isobenzofuran **6**, originating from 3,5-dimethoxy-benzyl alcohol via a three-step protocol with a combined yield of 42%.^17,18^

Prior to exploring proper conditions for the Diels–Alder key step for addressing the tetracene backbone, the installation of the tertiary alcohol was investigated by using fragment **7**. A Rubottom oxidation with MMPP as the oxidizing agent was employed.^19^ We observed that MMPP provided higher yields than other oxidants, such as Davis’ oxaziridine or *m*CPBA, and resulted in the formation of diastereomerically pure ketol **10** in 79% yield (Scheme 2).

**Scheme 2:**
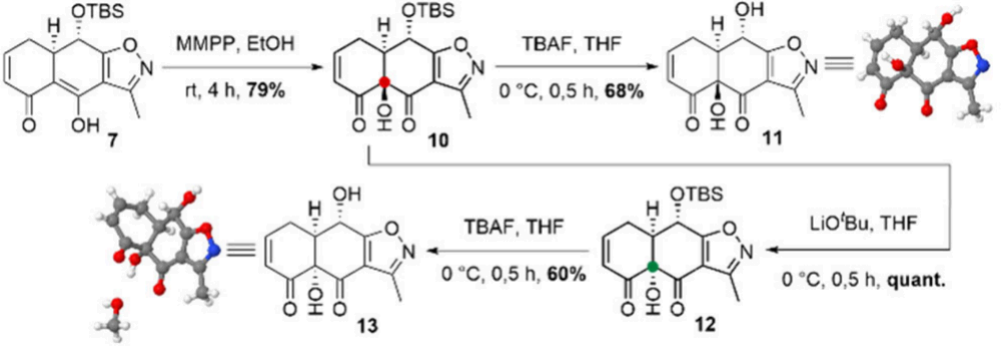
Formation of the Tertiary Alcohol and Its Inversion through Ketol Rearrangement

For an assignment of the absolute configuration of the tertiary alcohols, ketol **10** was subjected to TBS-deprotection conditions and the thus obtained diol **11** analyzed via X-ray diffraction. The crystallographic studies confirmed an undesired (*R*) configuration for the newly introduced tertiary alcohol. This outcome was found to be in agreement with previous observations made by the Nicolaou and Myers laboratories during their efforts to access various tetracycline derivatives.^14,20^ Lastly, the configuration of the tertiary alcohol was inverted via a base-catalyzed α-ketol rearrangement.^21^

Originally reported in 1895 by de Bruyn and Ekenstein,^22^ the α-ketol rearrangement (also known as the acyloin rearrangement) is defined as a concerted [1,2]-alkyl shift induced primarily in tertiary ketols either by bases, Brønsted and Lewis acids, or heat.^23,24^

Here, the base-catalyzed α-ketol rearrangement^21^ resulted in the successful adjustment of the C8a center of ketol **10**, affording a (*S*)-configured tertiary alcohol **12** with high yields. The underlying mechanism of the transformation is highlighted in Scheme 3. Under the action of catalytic amounts of LiO^*t*^Bu, an α-ketol rearrangement is triggered in ketol **10** by first deprotonating the tertiary alcohol to form the corresponding alkoxide species **14**. The alkoxide then readily rearranges to form an annulated 5/7/5-tricyclic intermediate **15**, followed by a 90° rotation of C1-carbonyl. The second ketol rearrangement takes place and generates compound **12**, in which the two carbonyl groups are now *anti*-oriented, with the more favorable orientation representing the driving force of the reaction.

**Scheme 3:**
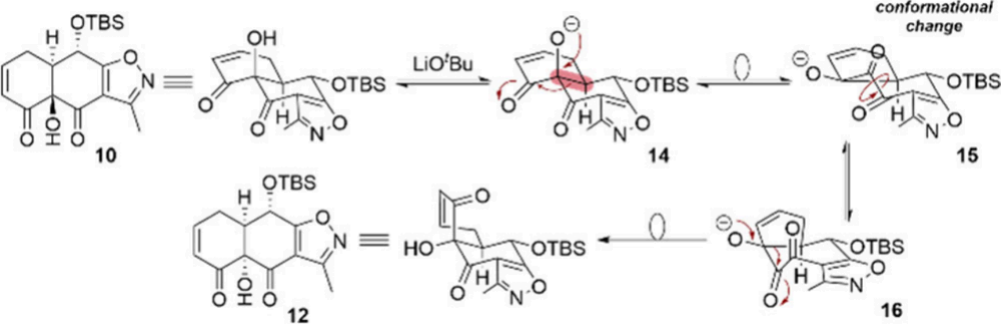
Mechanism of the LiO^*t*^Bu-Induced α-Ketol Rearrangement in Ketol **10**

In addition to the obtained crystallographic data for the two ketols **10** and **12**, NMR spectroscopic data were also used for the structure determination. While the recorded chemical shifts of the two products differ only slightly, a strong indication of the successful outcome of the reaction lies in the chemical shifts of the C8a atoms. Recorded at 75.4 ppm (CDCl_3_), the C8a atom of ketol **10** experiences a low-field shift after performing the rearrangement toward **12**, with the newly recorded value for C8a lying at 79.6 ppm (CDCl_3_). These observations were found to be largely in accordance with literature data.^14^

With the synthesis of fragments **6** and **7** successfully completed, we turned our attention on the synthesis of the tetracene skeleton via the envisioned Diels–Alder reaction. Then, the focus was directed toward the [4 + 2] cycloaddition. An account from the Myers laboratory describes the potential use of this transformation for the construction of tetracycline architectures.^25–27^ However, with the confrontation of poor yields and selectivity for the investigated model reaction, this strategy was abandoned, shifting the focus toward the now well-established Michael–Dieckmann approach.^13^

For the *endo*-selective Diels–Alder reaction,^28,29^ a reliable generation and immediate conversion of the highly reactive furan species was of crucial importance, with screenings primarily focusing on two aspects: (I) an efficient deprotonation of phthalide **6** and (II) trapping the generated enolate with various silyl-protecting groups.^30^

At first, LDA^30–32^ was used for the deprotonation of **17**. While the use of TESCl and TBSCl failed to trap the generated anion,^33^ the use of TMSCl only managed to promote a Michael addition. The use of NaHMDS and TBSCl also failed to provide the desired transformation. However, when the base was changed to KHMDS, a successful outcome was achieved in combination with TBSCl as a trapping agent (Scheme 4).

**Scheme 4:**
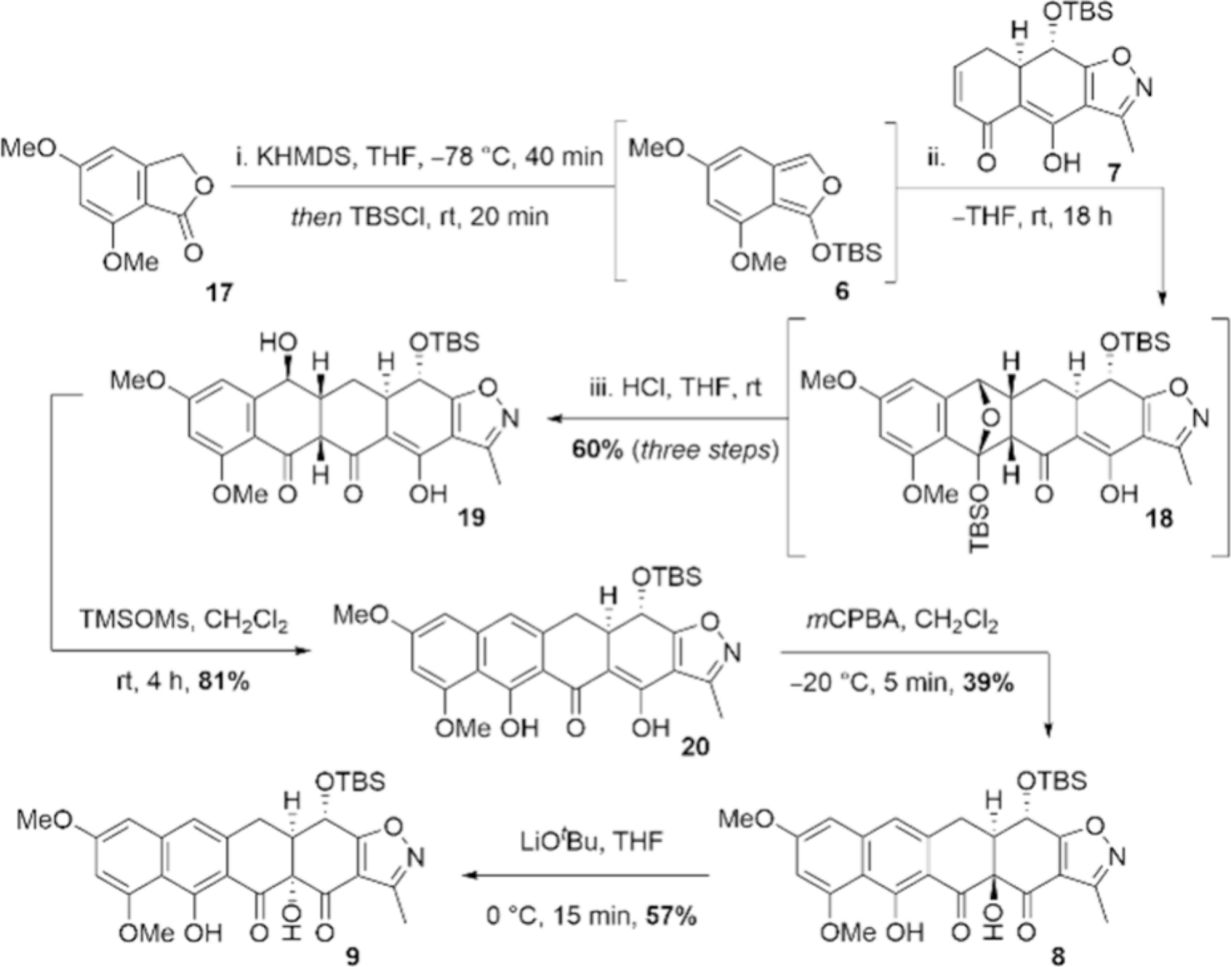
Overview of the Diels–Alder Key Step along with the Derivatization of the C12a Position in the Synthesis of Demethylpremithramycinone **3**

The isolated product was then quickly converted further under the action of a dilute acid to provide diastereomerically pure pentacycle **19** in a combined yield of 60%.

With the preparation of precursor **19** completed, a series of linear transformations were further employed in order to provide access to an advanced intermediate to natural product **2**. At first, the aromatization of the C ring was achieved via an elimination reaction.^33^ The employed transformation proceeded smoothly to give the advanced reaction intermediate **20** with an 81% yield.

For the late-stage generation of the C12a-positioned tertiary alcohol (ketol **21**), previously established conditions relying on the use of MMPP were investigated. However, while the synthesis of ketol **10** proceeded smoothly by this means, advanced ketol **8** could only be obtained after treating precursor **20** with *m*CPBA. The very short reaction time was found to be crucial in achieving reproducible results. Thus, the direct Rubottom oxidation of **20** was successfully completed with a moderate yield of 39%, with the outcome being comparable to previously reported ones for similar architectures.^34^ Furthermore, ketol **8** could be purified only by means of RP-HPLC, as other chromatographic methods led to degradation.

The undesired configuration of the freshly generated C12a-alcohol was then converted as described via an α-ketol rearrangement, with the conditions established previously in the synthesis of decalin **12**. The conversion of more demanding tetracycline-derived **8** proceeded, in contrast to decalin **12** with only a moderate yield of 57%, however. Furthermore, since the transformation only requires the use of a catalytic amount of LiO^*t*^Bu, the presence of an acidic phenol at C11 demanded the use of an increased amount of base (1.3 equiv) in order to initiate the rearrangement.

Since an accurate and irrefutable determination of the absolute configuration of ketols **8** and **9** has proven to be difficult by spectrometric and spectroscopic means, a NMR-comparative study in analogy to that performed for ketols **10** and **12** was considered instead. The observed low-field shift of the C12a signal in ketol **9**, the product of the rearrangement reaction (79.8 ppm for **9**, from an initial 76.3 ppm for **8**; spectra recorded in CDCl_3_), reinforced the assumptions made previously with regard to the outcome of the installation of the tertiary alcohol at the annulation position of the A/B rings. Thus, ketol **9** was obtained as the last advanced precursor to demethylpremithramycinone **2**, with the remaining transformations required for accessing the natural product concerning the cleavage of the protection groups as well as the scission of the isoxazole ring to furnish the 1,3-dicarbonyl motif at the A ring.

Despite the unfinished preparation of the originally envisioned target, the explored path managed to prove its potential on account of two fulfilled premises: (I) through a rapid and facile construction of the naphthacene core bearing most of the required final decorations via a [4 + 2]-cycloaddition key step and (II) by the ability to generate both (*R*)- and (*S*)-configured tertiary alcohols at the C12a position starting from deoxy precursors via the direct oxidation/α-ketol rearrangement strategy.

## Supplementary Material



## Data Availability

The data underlying this study are available in the published article and its Supporting Information.

## Notes

Experimental procedures and analytical and spectral data for new compounds (PDF)
