# Supplementary material for: α‑Ketol Rearrangement for Accessing Tetracyclic Natural Products
Source: Org Lett. 2025 Jul 22;27(30):8154–7. doi: 10.1021/acs.orglett.5c02235 (PMC12322950; doi:10.1021/acs.orglett.5c02235)
Supplement: Supplementary file 1 [file ol5c02235_si_001.pdf]

Supporting Information for

The α-ketol rearrangement for accessing tetracyclic natural products

Alexandru Sara[1], Ulrike Eggert[1] and Markus Kalesse*, [1,2]

[1] Institute of Organic Chemistry, Gottfried Wilhelm Leibniz Universität Hannover, 30167 Hannover, Germany

[2] Centre of Biomolecular Drug Research (BMWZ), Wilhelm Leibniz Universität Hannover, 30167 Hannover, Germany

Contents

1 Synthetic approach towards eastern fragment **7** **2**
2 General Methods **3**
3 Experimental procedures and analytical data **5**
  3.1 Synthesis of ketol **12** **5**
  3.2 Synthesis of western fragment **17** **14**
  3.3 Synthesis of tetracycline precursor **9** **16**
4 References **20**
5 Spectra and crystallographic parameters **22**
  5.1 Spectra **22**
  5.2 Crystallographic parameters **51**

1 Synthetic approach towards eastern fragment 7

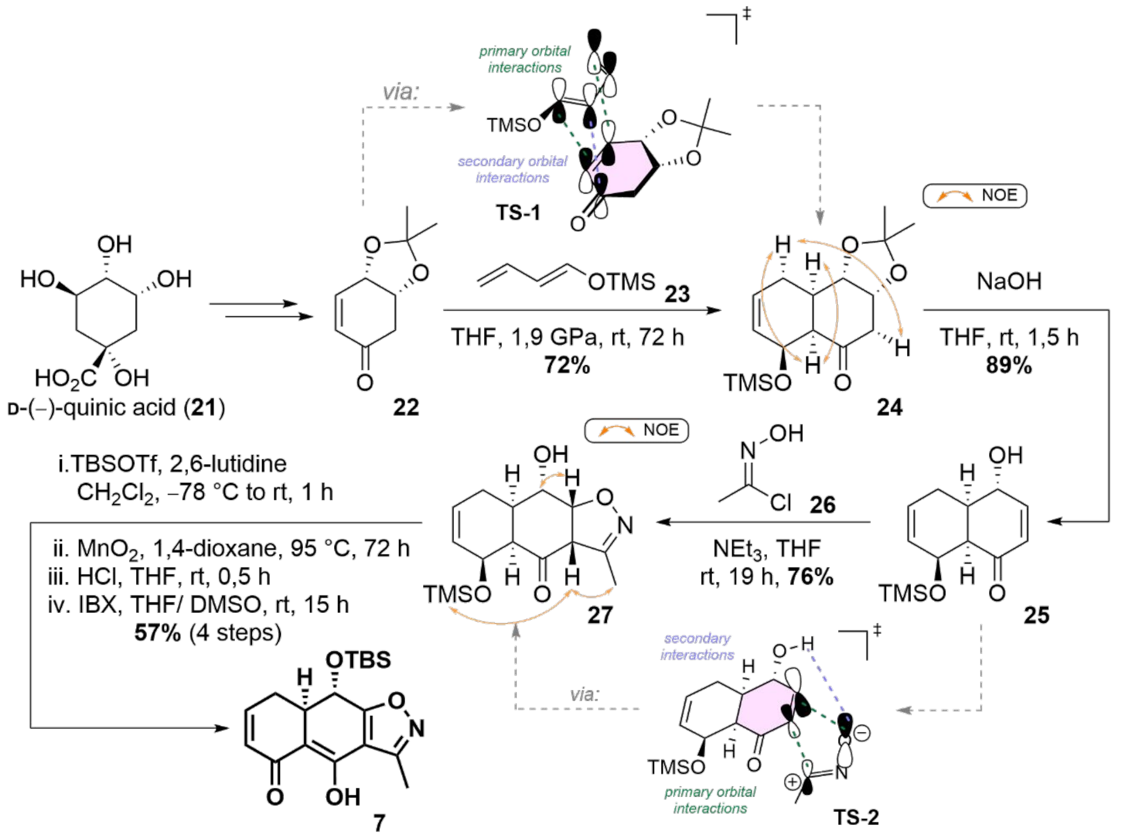

The preparation of fragment **7** commenced from D-(−)-quinic acid (**21**) and generated enone **22**.[1-4] A [4+2]-cycloaddition between enone **22** and literature-known silyloxybutadiene **23**[5] furnished compound **24** with its stereochemical outcome controlled by the concave/convex structure of the acetonide. By exposing the concentrated solutions of the two starting materials **22** and **23** in THF to pressures of up to 1,9 GPa, the exclusive formation of diastereomerically pure decalin **24** was observed in satisfactory yields. Following the cycloaddition reaction, decalin **24** was converted *via* an elimination reaction to the bicyclic enone **25**. For the generation of the isoxazole motif, a Huisgen 1,3-dipolar cycloaddition with nitrile oxide was chosen. For the *in-situ* generated nitrile oxide dipole, an approach originally proposed by Hyls et al.[6] was adapted in order to provide access to chloroxime **26**. By performing the synthesis of **26** in CH_2Cl_2, the freshly prepared reagent could be directly used for the following transformation, making the need for an aqueous work-up as well as the handling of potentially explosive precursors fully obsolete. The presence of *N*-hydroxysuccinimide in the reaction matrix was not found to influence the outcome of the cycloaddition step in any way. Thus, the envisioned [3+2]-cycloaddition with precursor **25** as the dipolarophile proceeded smoothly in the presence of NEt_3, resulting in the formation of tricyclic product **27** as a sole regio- and diastereomer.[7] In analogy to the observations made by Adembri et al.[8] during their preparation of several isoxazolines *via* a regio- and diastereoselective 1,3-dipolar cycloaddition between nitril oxides and 2-cyclopenten-1-ones, the secondary alcohol at *C*4 is considered to play a crucial role in

the stereochemical outcome of the reaction. Favoring the approach of the 1,3-dipole from the convex back-side through hydrogen bond formation, the *C*4-alcohol moiety ensures a strong diastereofacial selectivity in the formation of a concave-convex-arranged reaction product **27**. Furthermore, by tethering the dipole, the *C*4-alcohol is considered to play a crucial role in ensuring a regioselective process.
In order to generate the required isoxazole motif from the corresponding isoxazoline, an additional oxidation MnO_2-mediated step had to be performed.[9] Since the required conditions for this step might have led to an undesired oxidation of the secondary alcohol at *C*4, the protection of the functionality was required prior to the aromatization step. Lastly, upon cleaving the TMS-protection group orthogonally to the previously installed TBS-group and the oxidation of the thus released allylic alcohol at the *C*8-position, the preparation of *C*8a-deoxy fragment **7** was successfully completed on a multigram scale over eleven linear steps. The overall yield of these seven steps towards fragment **7** are 28%.

2 General methods

reagents, dry solvents and general reaction conditions: All used reagents and solvents, if not mentioned otherwise, were purchased from common chemical vendors (e.q. acros, abcr, tci) in the highest purity available. The use of technical grade solvents, as well as the purity of technical grade reagents are always indicated in the procedures. Deionized water was used for washing and extraction steps.
Most dry solvents were either purchased as such from common vendors, or were dried as needed by SPS (*solvent purification system*; SPS-800, MBRAUN®). Dry THF, CH_2Cl_2, Et_2O and NEt_3 were freshly distilled under an inert atmosphere (Ar) by using known protocols.[10]
Room temperature is defined as the ambient temperature at which the reactions were performed without external cooling, or heating, and ranges between 18.5 °C and 23.5 °C.

handling of air- and moisture-sensitive compounds: All air- and moisture-sensitive reactions were performed exclusively under an argon-atmosphere in suitable flame-dried glassware by using the Schlenk technique. Schlenk-flasks were used when needed and in accordance with available capacities. Regular round-bottom flasks, equipped with Teflon®- or silicon-septa however were also successfully used in handling sensitive reactions.

thin-layer chromatography: Thin-layer chromatography on silica-coated aluminium plates (25 x 25 cm; MACHEREY-NAGEL®; cut to size as needed) was used as a routine-method for checking the progress of reactions, as well as for the characterization of pure compounds. Detection of the probes was achieved both by UV-irradiation of the plate (254 nm), as well as staining with common reagents such as vanillin-stain or the cerium-molybdate based Seebach's reagent. Vinylogous acid-bearing compounds could be successfully stained by using a 0.1M aqueous ferric chloride-solution. Furthermore, all synthesized pentacyclic precursors have been found to show yellow-green fluorescence upon irritation with 254 nm light, this phenomenon coinciding with the emission of the TLC-plate. For a proper recognition of these compounds, a 365 nm UV-lamp was used instead.

flash column chromatography: Separation of mixtures was almost exclusively achieved *via* flash column chromatography. The crude products were purified on suitable glass-columns with a SiO_2-stationary phase (0.04 – 0.053 mm), the eluents being indicated within each individual procedure.

HPLC: High-performance liquid chromatography (Agilent Tech ProStar solvent delivery systems with one incorporated 4000 psi/ 200 ml pressure module) was employed for purifying advanced polar intermediates. The separation was carried out on a Dr. Maisch GmbH Reprosphere 100 column (10 µm, 250 x 20 mm; UV-detection at 254 nm and 310 nm) with var. H_2O/ MeCN-mixtures. The injected volumes (Knauer Smartline valve drive) alongside further parameters are individually indicated within the procedures. The fractions were automatically collected by a Cetac Autosampler ASX-7400 and the chromatograms analyzed with the PrepCon-software (*version 5.06.039*; SCPA GmbH).

solvent removal: Solvent-evaporation under reduced pressure was carried out by using rotary evaporators (HEILDOLPH; var. models) equipped with membrane pumps (VACUUBRAND®; var. models), the temperature being constantly kept at 40 °C and the pressure adjusted in accordance with the solvents used.

melting points* (m.p.)*: The determination of melting points for all isolated crystalline precursors was carried out by an automatic capillary-melting point apparatus (MPA100 *by* SRS®), when sufficient quantities of material were available. In the instances where recrystallization was considered as a purifying method, the used solvents are indicated alongside the measured melting points.

optical activity* ($[\alpha]^T$)*: For the measurement of optical rotations, a P8000-polarimeter (KRÜSS®) with temperature sensor was used. The samples were dissolved in spectroscopy-grade solvents (common vendors), the angles determined three times and the measured values corrected. The used solvent, the measuring-temperature T, as well as the sample-concentrations (mg/ml) are given for each measurement.

mass spectrometry: High-resolution ESI-spectra (Micromass LCT Premier with HPLC-module Arc 2695; WATERS™) were measured for all synthesized compounds. The samples were diluted with spectroscopy-grade solvents prior to injection.

NMR-spectroscopic data: NMR-analyses were carried out by using various spectrometers from BRUKER® (Ultrashield 400 MHz (ULS400) or Ascend 400 MHz (ASC400)), the reported spectra being evaluated with the Mnova-software (*version 14.3.1*; MESTRELAB RESEARCH®). $CDCl_3$ was purchased from commercial suppliers and used as received. The residual solvent peaks were used for referencing the spectra; for ^1H-spectra: 7.26 ppm and for $^{13}C\{^1H\}$-spectra: 77.2 ppm.[11] The acronyms and abbreviations used to describe the signals are: m (multiplet; when an assignment cannot be carried out unambiguously), *s* (singlet), *br. s* (broad singlet), *d* (doublet), *t* (triplet), *q* (quartet), *dd* (doublet of doublets), *dt* (doublet of triplets), *ddd* (doublet of doublet of doublets), *ddt* (doublet of doublet of triplets), *td* (triplet of doublets).

atom-labeling in context of NMR-analysis: The numbering system used in the assignment of both ^1H- and $^{13}C\{^1H\}$-signals is arbitrary and based on the numbering traditionally used for tetracycline architectures. While carbon-atoms are numbered with arabic numerals, geminal hydrogen-atoms are labeled with letters.

X-ray crystallographic data: For the analysis of compound **19** a suitable crystal (0.18×0.40×0.82 mm^3) was selected and mounted on a SMART X2S single diffractometer. The structure was solved with the ShelXT 2014/5 structure solution program using the direct solution method. The model was refined with version 2018/3 of ShelXL 2018/3 using Least Squares minimization.
For the analysis of compound **21** a suitable crystal (0.60×0.40×0.04 mm^3) was selected and mounted on a 18 mm CryoLoop (20 micron, 0.2-0.3 mm, Hampton Research) on a XtaLAB AFC12 (RINC): Kappa single diffractometer. The crystal was kept at a steady T = 99.99(10) K during data collection.

The structure was solved with the ShelXT 2018/2 structure solution program using the Intrinsic Phasing solution method. The model was refined with version 2019/3 of ShelXL 2019/3 using Least Squares minimization.

high-pressure set-up: High-pressure Diels–Alder reactions were performed by subjecting reaction solutions in sealed Teflon®-tubes (Ø 1.25 cm) to pressures of up to 15.000 bar. A special set-up (Andreas Hofer Hochdrucktechnik GmbH; model-nr.: HP14), consisting of a cylindrical reaction chamber filled with silicone oil and equipped with an external hydraulic pump (ext.-pressure up to 450 bar; conversion-rate for calculating internal pressures 1 : 33.64 bar) was employed for this task.

hating of reactions: An oil bath was used for all reactions required heating.

3 Experimental procedures and analytical data

3.1 Synthesis of ketol **12**

Enone **22**

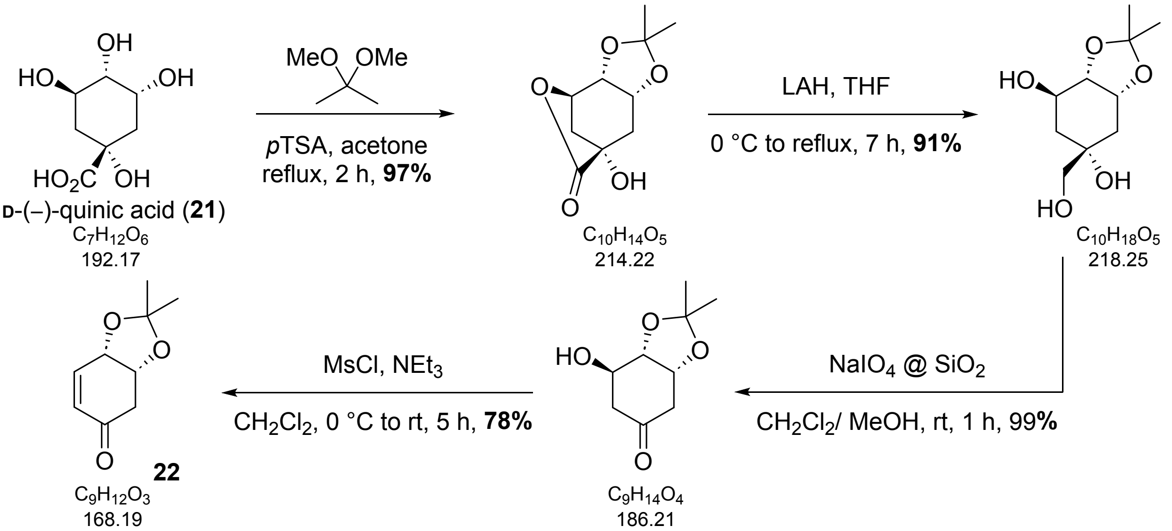

esterification **[1]*:*** 19.5 g (101 mmol, 1.0 equiv.) D-(–)-quinic acid (**21**) along with 45.0 ml (366 mmol, 3.6 equiv.) 2,2-dimethoxypropane and 1.95 g (10.3 mmol, 10 mol-%) *para*-toluenesulfonic acid monohydrate were dissolved in 675 ml technical grade acetone in a 1-l one-neck round-bottom flask. The formed cloudy mixture was refluxed for two hours (**R**$_f$ = 0.54 (PE/ EtOAc = 1:1 (v/v))) after which it was neutralized with 3.0 ml wet NEt_3. The formed pale yellow solution was then concentrated under reduced pressure to yield an off-white crystalline powder (21.1 g, 90.6 mmol, **97%**), which was used in the following step without further purification.

[1] The synthesis was carried out by following a protocol described by Kawashima et al. with the recorded spectrometric and spectroscopic data being in accordance with those previously reported by the authors.[12]

LAH-reduction [2]**:** A flame-dried 500-ml three-neck round-bottom flask, equipped with a condenser was charged with 4.71 g (124 mmol, 1.3 equiv.) lithium aluminium hydride, suspended in 263 ml dry THF. Upon cooling of the mixture to 0 °C, a solution consisting of 21.1 g (90.6 mmol, 1.0 equiv.) of the previously prepared ester, dissolved in 123 ml dry THF, was added *via* cannula. The formed cloudy suspension was refluxed for seven hours ($\mathbf{R_f}$ = 0.54 (EtOAc)), after which it was cooled again to 0 °C. Any unreacted alanate-residues were then neutralized by the careful addition of 4.5 ml of water, followed by 4.5 ml of 15% aqueous NaOH-solution and further 4.5 ml of water, and the mixture allowed to warm to room temperature. The precipitated aluminium salts were bonded with Na_2SO_4, filtered off *via* a sintered glass funnel and the filtrate concentrated *in vacuo* to yield a triol-intermediate (19.5 g, 89.3 mmol, **91%**) as a white crystalline powder, which was further used without any purification.

periodate cleavage [3]**:** A 2-l one-neck round-bottom flask was charged with the previously prepared triol (19.5 g, 89.3 mmol, 1.0 equiv.) and a mixture consisting of 595 ml CH_2Cl_2 and 30.0 ml MeOH was added. For the preparation of the SiO_2-supported $NaIO_4$-mixture, 134 g SiO_2 were gradually added to a saturated aqueous solution of 28.6 g (134 mmol, 1.5 equiv.) $NaIO_4$ in 62.0 ml boiling water. The thus formed reagent-matrix was added portion wise (*exothermic reaction!*) to the CH_2Cl_2/ MeOH-solution of the triol and the suspension allowed to stir for one hour ($\mathbf{R_f}$ = 0.50 (PE/ EtOAc = 5:3 (v/v))). Upon completion the solids were filtered, washed with warm 5% EtOH in CH_2Cl_2 and the filtrate concentrated under reduced pressure to yield 16.5 g (88.6 mmol, **99%**) of the aimed title compound as a pale yellow oil, which was further used without any purification.

elimination [4] (synthesis of enone **22**): 15.2 g (81.6 mmol, 1.0 equiv.) of the previously prepared β-ketol were charged in a flame-dried 1-l one-neck round-bottom flask and dissolved in 513 ml dry CH_2Cl_2. The solution was then cooled to 0 °C upon which dry NEt_3 (34.2 ml, 245 mmol, 3.0 equiv.) and MsCl (7.6 ml, 98.3 mmol, 1.2 equiv.) were added. The formed reaction mixture was then stirred under an inert atmosphere for five hours at room temperature. Upon completion ($\mathbf{R_f}$ = 0.51 (PE/ EtOAc = 3:2 (v/v))), 150 ml $NaHCO_3$-sol. (sat., aq.), followed by 300 ml of water were added and the mixture charged in a separatory funnel. The organic phase was separated and the aqueous phase extracted two times with a total of 300 ml CH_2Cl_2. The combined organic phases were then dried over Na_2SO_4 and concentrated *in vacuo*. Purification of the raw material *via* flash column chromatography (SiO_2, PE/ EtOAc = 2:1 (v/v)) finally yielded enone **22** (10.7 g, 63.3 mmol, **78%**) as a colorless oil

[2] *The synthesis was carried out by following a protocol described by Trost et al. with the recorded spectrometric and spectroscopic data being in accordance with those previously reported by the authors.[3]*

[3] *The synthesis was carried out by following a protocol described by Wang et al. with the recorded spectrometric and spectroscopic data being in accordance with those previously reported by the authors.[4]*

[4] *The synthesis was carried out by following a protocol described by Kawashima et al. with the recorded spectrometric and spectroscopic data being in accordance with those previously reported by the authors.[12]*

Ketene acetal **23** [5]

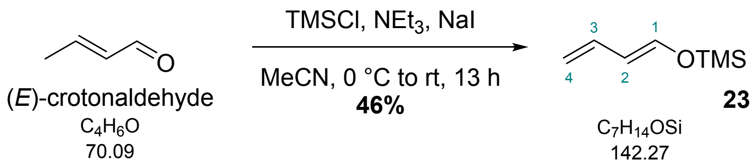

(*E*)-crotonaldehyde (13.0 g, 15.4 ml, 186 mmol, 1.0 equiv.) and dry NaI (44.5 g, 297 mmol, 1.6 equiv.) were dissolved in a flame-dried three-neck round-bottom flask in 185 ml dry acetonitrile. The orange-colored suspension was then cooled to 0 °C and dry NEt_3 (41.4 ml, 297 mmol, 1.6 equiv.) added. Lastly, TMSCl (35.3 ml, 278 mmol, 1.5 equiv.) was added and the milky solution allowed to stir under an inert atmosphere at room temperature for 13 hours. Upon completion (**R_f** = 0.92 (PE/ EtOAc = 9:1 (v/v))),the mixture was diluted with 150 ml *n*-pentane. The MeCN-phase was then extracted with *n*-pentane (3x150 ml) after which it was discarded. The combined solutions were then washed with 250 ml $NaHCO_3$-sol. ($^1/_2$-sat., aq.), dried over Na_2SO_4 and concentrated under reduced pressure. Purification of the crude *via* vacuum-distillation ($T_{ex.}=72$ °C, $T_{int.}=55$ °C; P = 40 mbar) afforded diene **23** (12.2 g, 86.0 mmol, **46%**) as a colorless oil. The recorded spectroscopic data match those reported previously.[5]

Cycloaddition product **24**

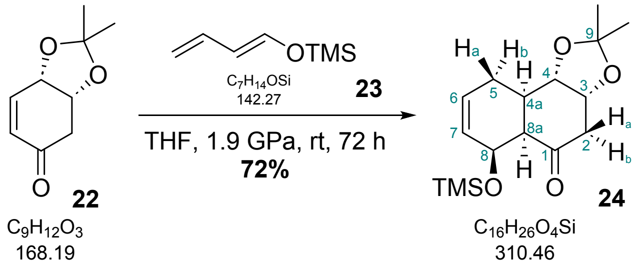

Enone **22** (5.45 g, 32.4 mmol, 1.0 equiv.) and ketene acetal **23** (11.5 g, 81.0 mmol, 2.5 equiv.) were dissolved in 10.1 ml dry THF in a Teflon® sealed-tube. The cycloaddition reaction was then carried out for 72 hours at room temperature under 1.9 GPa. Upon completion the tube was unsealed, the volatiles removed *in vacuo* and the oily residue charged on a flash chromatography column (SiO_2). Gradual elution (PE→PE/ EtOAc = 9:1→5:1 (v/v)) afforded decalin **24** (7.29 g, 23.5 mmol, **72%**) as a colorless oil.

R_f = 0.83 (PE/ EtOAc = 3:1 (v/v));

$[\alpha]_D^{22.3}$ = +110.53° ($CHCl_3$, *3.80*);

HR-ESI-MS: $[C_{16}H_{26}O_4SiNa]^+$: calc. m/z = 333.1498, found m/z = 333.1500;

^{1}H-NMR (400 MHz; $CDCl_3$): δ [ppm] = 5.80–5.84 (m, **1H**), 5.74–5.78 (m, **1H**), 4.61 (dd, *J* = 10.1, 7.5 Hz, **1H**), 4.41–4.43 (m, **1H**), 4.37–4.40 (m, **1H**), 2.78 (dd, *J* = 16.3, 6.4 Hz, **1H**), 2.51 (dd, *J* = 16.3, 9.1 Hz, **1H**), 2.45 (t, *J* = 4.8, 4.8 Hz, **1H**), 2.41–2.42 (m, **1H**), 2.37 (dd, *J* = 10.4, 5.0 Hz, **1H**), 2.23–2.31 (m, **1H**), 1.50 (s, **3H**, CH_3), 1.35 (s, **3H**), 0.07 (s, **9H**);

---

[5] *The synthesis was carried out by following the protocol described by Böse et al. with the recorded spectroscopic data being in accordance with those previously reported by the authors.*[5]

^{13}**C-NMR** (101 MHz; $CDCl_3$): δ [ppm] = 211.5 (**1C**), 128.0 (**1C**), 127.9 (**1C**), 108.4 (**1C**), 74.4 (**1C**), 72.2 (**1C**), 64.8 (**1C**), 49.4 (**1C**), 44.7 (**1C**), 32.8 (**1C**), 27.4 (**1C**), 26.4 (**1C**), 24.2 (**1C**), –0.3 (**3C**).

Decalin **25**

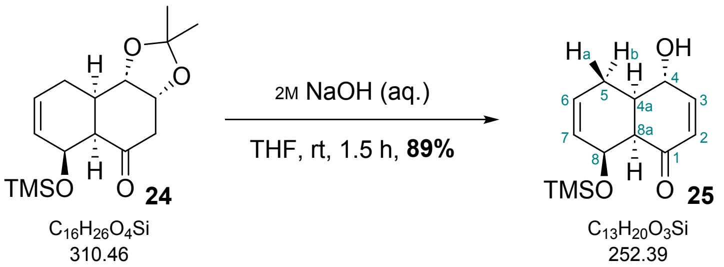

Decalin precursor **24** (7.29 g, 23.5 mmol, 1.0 equiv.) was dissolved in a 100-ml one-neck round-bottom flask under ambient conditions in 47.0 ml THF and treated with 2.4 ml (2.75 mmol, 20 mol-%) aqueous NaOH-solution (2M). The reaction was then stirred for 1.5 hours at room temperature and terminated by the addition of 50 ml of water. After extraction with MTBE (3x100 ml), the organic phase was dried over Na_2SO_4, the volatiles removed under reduced pressure and the residue purified *via* flash column chromatography (SiO_2, PE/ EtOAc = 4:1 (v/v)) to afford product **25** (5.29 g, 20.9 mmol, **89%**) as a colorless oil.

$\mathbf{R_f}$ = 0.63 (PE/ EtOAc = 1:1 (v/v));

$[\alpha]_D^{22.1}$ = +337.24° ($CHCl_3$, *3.22*);

HR-ESI-MS: $[C_{13}H_{20}O_3SiNa]^+$: calc. m/z = 275.1079, found m/z = 275.1081;

^{1}**H-NMR** (400 MHz; $CDCl_3$): δ [ppm] = 6.90 (dd, *J* = 10.2, 1.7 Hz, **1H**), 5.98 (ddd, *J* = 10.2, 2.2, 0.9 Hz, **1H**), 5.78–5.80 (m, **2H**), 4.82 (t, *J* = 8.2, 8.2 Hz, **1H**), 4.33 (td, *J* = 4.4, 4.3, 1.6 Hz, **1H**), 2.68 (t, *J* = 4.9, 4.9 Hz, **1H**,), 2.54–2.60 (m, **1H**), 2.37 (dt, *J* = 10.9, 5.8, 5.8 Hz, **1H**), 2.12–2.19 (m, **1H**), 1.79 (d, *J* = 7.1 Hz, **1H**), 0.00(s, **9H**);

^{13}**C-NMR** (101 MHz; $CDCl_3$): δ [ppm] = 200.9 (**1C**), 154.4 (**1C**), 130.0 (**1C**), 128.6 (**1C**), 127.1 (**1C**), 67.4 (**1C**), 64.4 (**1C**), 50.0 (**1C**), 40.0 (**1C**), 24.9 (**1C**), 0.1 (**3C**).

Chloroxime **26** [6] and isoxazoline **27**

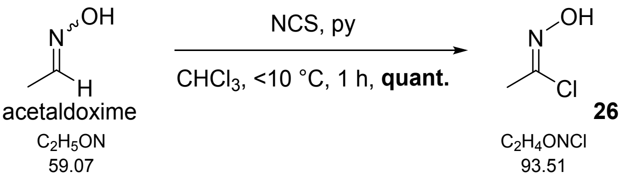

A 100-ml one-neck round-bottom flask was charged with 39.0 ml dry $CHCl_3$, 3.8 ml (62.0 mmol, 1.0 equiv.) acetaldoxime and pyridine (0.1 ml, 1.24 mmol, 20 mol-%) and the solution cooled to 0 °C. NCS (8.29 g, 62.1 mmol, 1.0 equiv.) was then added portion wise and the mixture stirred for one hour while the temperature was carefully maintained bellow 10 °C. [7] Upon completion, the reaction mixture was transferred *via* steel cannula to the cycloaddition step.

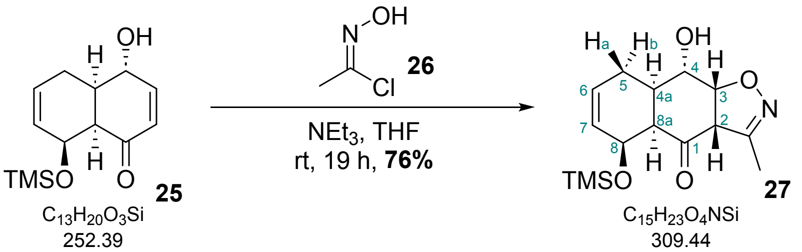

For the installation of the isoxazoline moiety, a flame-dried 100-ml one-neck round-bottom flask was charged under an inert atmosphere at 0 °C with 22.0 ml dry THF, to which 4.47 g (17.7 mmol, 1.0 equiv.) decalin **25** and 9.2 ml (65.6 mmol, 3.7 equiv.) dry NEt_3 were added. The mixture was then treated with a freshly prepared solution of **26** (3.5 equiv.) and the reaction allowed to stir at room temperature for 19 hours. Upon completion, the reaction was diluted with 50 ml of water and transferred to a separatory funnel. After extraction with MTBE (3x50 ml) the organic phases were dried over Na_2SO_4, concentrated *in vacuo* and the residue purified *via* flash column chromatography (SiO_2, CH_2Cl_2 [8]; *then* PE/ EtOAc = 4:1 (v/v)). Product **27** (4.15 g, 13.4 mmol, **76%**) was isolated as a single diastereo- and regioisomer in form of a white microcrystalline powder.

$\mathbf{R_f}$ = 0.40 (PE/ EtOAc = 3:2 (v/v));

m.p. = 122 - 124 °C;

$[\boldsymbol{\alpha}]_{\mathbf{D}}^{\mathbf{22.1}}$ = +280.70° ($CHCl_3$, *4.75*);

HR-ESI-MS: $[C_{15}H_{23}O_4NSiNa]^+$: calc. m/z = 332.1294, found m/z = 332.1295;

^{1}H-NMR (400 MHz; $CDCl_3$): δ [ppm] = 5.85 (ddd, J = 10.1, 5.5, 2.1 Hz, **1H**), 5.74–5.82 (m, **1H**), 4.88 (dd, J = 10.2, 4.3 Hz, **1H**), 4.75 (dd, J = 11.9, 4.3 Hz, **1H**), 4.35–4.38 (m, **1H**), 3.66 (d, J = 10.3 Hz, **1H**), 2.70–2.72 (m, **1H**), 2.67 (d, J = 5.4 Hz, **1H**), 2.39 (dt, J = 11.0, 5.3, 5.3 Hz, **1H**), 2.12 (s, **3H**), 2.05 (td, J = 4.2, 4.0, 2.2 Hz, **1H**), 2.00–2.02 (m, **1H**), 0.07 (s, **9H**);

---

[6] *The synthesis was adapted after a protocol described by Hylse et al.*[6]

[7] *During the course of the reaction, the color of the solution changed from an initial dark grey to magenta.*

[8] *For the elution of 3,4-dimethyl-1,2,5-oxadiazole 2-oxide (*$\boldsymbol{R_f}$ *= 0.42 (PE/ EtOAc = 3:2 (v/v))), formed as a side-product.*[13]

^{13}**C-NMR** (101 MHz; $CDCl_3$): δ [ppm] = 205.1 (**1C**), 155.1 (**1C**), 128.1 (**1C**), 127.0 (**1C**), 80.5 (**1C**), 65.8 (**1C**), 65.3 (**1C**), 63.0 (**1C**), 52.2 (**1C**), 31.6 (**1C**), 23.7 (**1C**), 13.2 (**1C**), 0.3 (**3C**).

Eastern fragment 7

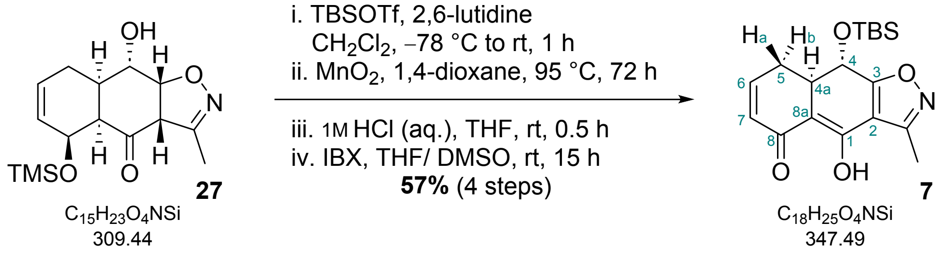

TBS-protection: In a flame-dried 100-ml one-neck round-bottom flask 3.45 g (11.1 mmol, 1.0 equiv.) of isoxazoline **27** were dissolved under an inert atmosphere in 32.0 ml dry CH_2Cl_2. The solution was then cooled to –78 °C, 2,6-lutidine (2.6 ml, 22.3 mmol, 2.0 equiv.) and TBSOTf (3.3 ml, 14.5 mmol, 1.3 equiv.) added and the reaction allowed to stir while maintaining the initial temperature. After one hour (**R_f** = 0.69 (PE/ EtOAc = 4:1 (v/v))) the cooling bath was removed, the reaction allowed to warm up to room temperature and diluted with 50 ml of water. Extraction with MTBE (3x50 ml), followed by drying over Na_2SO_4 and concentration under reduced pressure gave the desired TBS-protected intermediate (4.68 g, 11.1 mmol) as a pale yellow oil, which was used further without any purification.

aromatization: 2.05 g (4.84 mmol, 1.0 equiv.) of the previously prepared isoxazoline, dissolved in 32.0 ml 1,4-dioxane were charged under ambient conditions in a sealed tube and 5.26 g (48.4 mmol, 10.0 equiv.) MnO_2 (80 wt%; technical grade) added. The tube was then sealed and the reaction allowed to stir at 95 °C for 72 hours. Upon completion (**R_f** = 0.77 (PE/ EtOAc = 4:1 (v/v)); progress additionally monitored every 24 hours by ^{1}H-NMR [9]), the black slurry was filtered over a pad of Celite® (MTBE) and concentrated under reduced pressure to give 1.31 g (3.11 mmol) of the aimed crude isoxazole-bearing decalin as a pale yellow powder.

TMS-cleavage: The previously prepared isoxazole (1.31 g, 3.11 mmol, 1.0 equiv.), dissolved at room temperature in 124 ml THF was reacted with 24.9 ml (8.0 equiv.) aqueous HCl-solution (1M) for 30 minutes in a 250-ml one-neck round-bottom flask under ambient conditions (**R_f** = 0.50 (PE/ EtOAc = 4:1 (v/v))). The reaction was then neutralized by the addition of 30 ml saturated aqueous $NaHCO_3$-soution followed by the addition of 50 ml MTBE. The aqueous phase was then extracted with MTBE (3x50 ml). The combined ethereal organic phases were dried over Na_2SO_4 and concentrated under reduced pressure to yield 1.08 g (3.09 mmol) of the aimed crude allylic alcohol as a yellow oil.

IBX-oxidation (synthesis of **7**): In a flame-dried 100-ml one-neck round-bottom flask, 1.08 g (3.09 mmol, 1.0 equiv.) of the previously prepared crude allylic alcohol were dissolved under an inert atmosphere in 31.0 ml dry THF and 3.0 ml dry DMSO. To the formed solution were then added 2.60 g (9.27 mmol, 3.0 equiv.) IBX and the heterogenous mixture allowed to stir for 15 hours at room temperature. Upon completion, the orange-colored slurry was diluted with a small amount of MTBE and filtered over a pad of Celite®. Removal of the volatiles under reduced pressure yielded a thick, orange-colored oil, which was purified *via* flash column chromatography (SiO_2, PE/ EtOAc = 9:1 (v/v)). Eastern fragment **9** (0.98 g, 2.82 mmol, **58%** (4 steps)) was collected as golden-shimmery, yellow-colored crystalline flakes.

---

[9] *A low-field shift of the CH_3-group (400 MHz, $CDCl_3$; δ = 2.47 ppm from an original 2.14 ppm) was observed as a result of the aromatization of the system.*

R_f = 0.84 (PE/ EtOAc = 4:1 (v/v));

m.p. = 130 - 131 °C;

$[\alpha]_D^{19.4}$ = –12.12° ($CHCl_3$, *1.10*);

HR-ESI-MS: $[C_{18}H_{25}O_4NSiNa]^+$: calc. m/z = 370.1451, found m/z = 370.1445;

^{1}H-NMR (400 MHz; $CDCl_3$): δ [ppm] = 6.63 (ddd, *J* = 9.3, 6.2, 2.4 Hz, **1H**), 6.15 (dd, *J* = 9.9, 3.1 Hz, **1H**), 4.73 (d, *J* = 10.8 Hz, **1H**), 2.98–3.07 (m, **1H**), 2.85 (dt, *J* = 17.4, 6.7, 6.7 Hz, **1H**), 2.52 (s, **3H**), 2.13 (ddt, *J* = 17.8, 15.9, 2.9, 2.9 Hz, **1H**), 0.97 (s, **9H**), 0.28 (s, **3H**), 0.18 (s, **3H**);

^{13}C-NMR (101 MHz; $CDCl_3$): δ [ppm] = 182.7 (**1C**), 177.4 (**1C**), 173.5 (**1C**), 157.7 (**1C**), 141.3 (**1C**), 125.6 (**1C**), 113.2 (**1C**), 102.8 (**1C**), 71.7 (**1C**), 39.6 (**1C**), 29.8 (**1C**), 25.9 (**3C**), 18.3 (**1C**), 10.8 (**1C**), –4.3 (**1C**), –4.6 (**1C**).

Ketol **10**

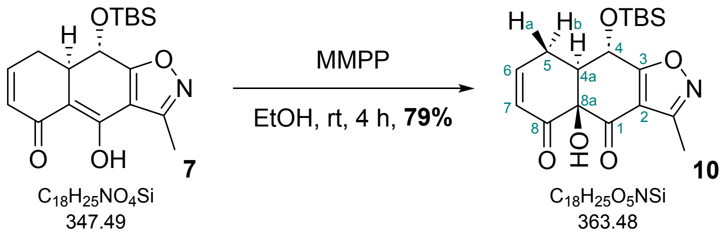

A 5-ml one-neck round-bottom flask was charged at room temperature with 125 mg (0.36 mmol, 1.0 equiv.) of **7** and 0.9 ml EtOH. 111 mg (80 wt%, 0.18 mmol, 0.5 equiv.) MMPP·6H_2O were added. The formed red-colored solution was stirred for four hours. Upon completion, the reaction was diluted with 3 ml of water, the organic components extracted with CH_2Cl_2 (3x10 ml) and the combined organic phases dried over Na_2SO_4. After the solvent was removed under reduced pressure, the residue was purified *via* flash column chromatography (SiO_2, PE/ EtOAc = 4:1 (v/v)) to yield ketol **10** (103 mg, 0.28 mmol, **79%**) as an orange-colored solid.

R_f = 0.44 (PE/ EtOAc = 3:2 (v/v));

m.p. = 139 - 142 °C;

$[\alpha]_D^{22.5}$ = –56.67° ($CHCl_3$, *3.00*);

HR-ESI-MS: $[C_{18}H_{25}O_5NSiNa]^+$: calc. m/z = 386.1400, found m/z = 386.1405;

^{1}H-NMR (400 MHz; $CDCl_3$): δ [ppm] = 7.09 (ddd, *J* = 10.4, 5.9, 1.6 Hz, **1H**), 6.14 (dd, *J* = 10.1, 2.3 Hz, **1H**), 5.14 (d, *J* = 8.8 Hz, **1H**), 4.04 (br. s, **1H**), 2.75–2.80 (m, **1H**), 2.61–2.72 (m, **2H**), 2.45 (s, **3H**), 0.97 (s, **9H**), 0.32 (s, **3H**), 0.23 (s, **3H**);

^{13}C-NMR (101 MHz; $CDCl_3$): δ [ppm] = 194.1 (**1C**), 188.1 (**1C**), 179.4 (**1C**), 158.4 (**1C**), 148.7 (**1C**), 128.7 (**1C**), 112.7 (**1C**), 75.4 (**1C**), 66.0 (**1C**), 49.3 (**1C**), 25.8 (**3C**), 25.1 (**1C**), 18.3 (**1C**), 10.7 (**1C**), –4.4 (**1C**), –4.7 (**1C**).

Rearranged ketol **12**

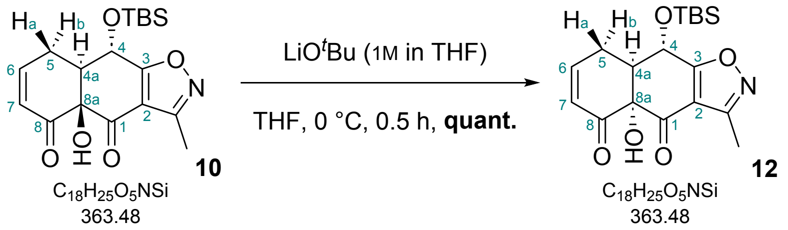

The previously prepared ketol **10** (50 mg, 0.14 mmol, 1.0 equiv.) was dissolved in 1.4 ml dry THF in a flame-dried 10-ml one-neck round-bottom flask and a LiO^{t}Bu-solution (1M in THF, 325 µl, 0.03 mmol, 20 mol-%) added at 0 °C. After stirring for 30 minutes, the mixture was diluted with 5 ml of water, extracted with MTBE (3x10 ml) and the combined organic phases dried over Na_2SO_4. Solvent removal under reduced pressure afforded the rearranged ketol **12** (49.2 mg, 0.14 mmol, **quant.**) as an off-orange powder. A purification of the product was not required.

$\mathbf{R_f}$ = 0.78 (PE/ EtOAc = 3:2 (v/v));

m.p. = 130 - 131 °C;

$[\alpha]_D^{26.0}$ = +145.88° ($CHCl_3$, *6.38*);

HR-ESI-MS: $[C_{18}H_{25}O_5NSiNa]^+$: calc. m/z = 386.1400, found m/z = 386.1402;

^{1}H-NMR (400 MHz; $CDCl_3$): δ [ppm] = 6.97–7.02 (m, **1H**), 6.19 (ddd, *J* = 10.3, 2.8, 1.2 Hz, **1H**), 4.94 (d, *J* = 8.8 Hz, **1H**), 2.93–2.95 (m, **1H**), 4.47 (s, **1H**), 2.88–2.93 (m, **1H**), 2.66 (dd, *J* = 19.3, 5.5 Hz, **1H**), 2.52 (s, **3H**), 0.95 (s, **9H**), 0.26 (s, **3H**), 0.16 (s, **3H**);

^{13}C-NMR (101 MHz; $CDCl_3$): δ [ppm] = 192.7 (**1C**), 189.6 (**1C**), 179.6 (**1C**), 157.8 (**1C**), 148.6 (**1C**), 129.0 (**1C**), 113.2 (**1C**), 79.6 (**1C**), 64.9 (**1C**), 51.2 (**1C**), 25.8 (**3C**), 24.5 (**1C**), 18.2 (**1C**), 10.8 (**1C**), –4.3 (**1C**), –4.8 (**1C**).

2.2 Synthesis of phthalide **17**

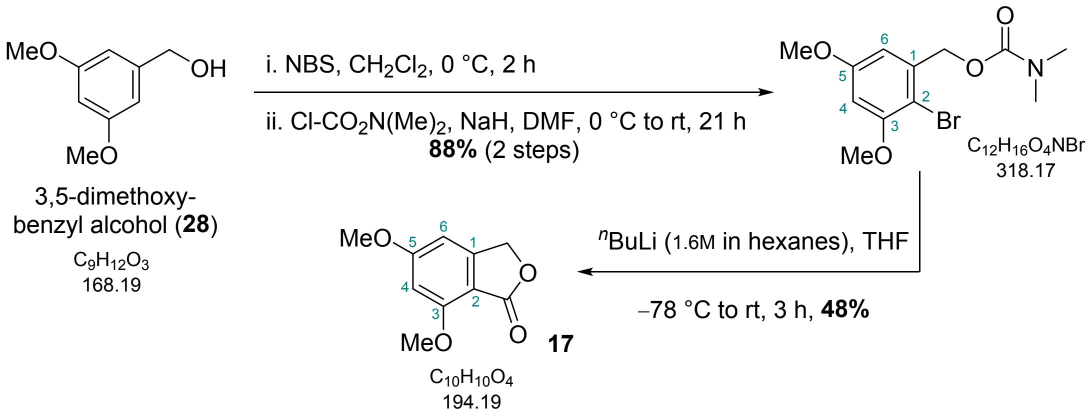

bromination* [10]*: 3,5-dimethoxybenzyl alcohol **28** (12.5 g, 74.3 mmol, 1.0 equiv.) was dissolved under ambient conditions in a 250-ml one-neck round-bottom flask in 203 ml CH_2Cl_2.and cooled to 0 °C. To the formed solution a total of 13.2 g (74.3 mmol, 1.0 equiv.) NBS were added in three portions over a period of 45 minutes. Upon addition the reaction was allowed to stir for a total of two hours (**R_f** = 0.27 (PE/ EtOAc = 7:3 (v/v))), after which the volatiles were removed under reduced pressure. The residue was then suspended in 100 ml of water and extracted with EtOAc (3x100 ml). The combined organic phases were dried over Na_2SO_4 and concentrated *in vacuo* to yield the aimed brominated intermediate (18.0 g, 72.9 mmol) as a white powder.

carbonylation: The previously prepared bromide (18.0 g, 72.9 mmol, 1.0 equiv.) was charged in a flame-dried 250-ml one-neck round-bottom flask and dissolved in 112 ml dry DMF. The solution was then cooled to 0 °C and 3.79 g (94.7 mmol, 1.3 equiv.) NaH (60 wt% in mineral oil) were carefully added (*vigorous gas formation!*). After the addition was completed, the reaction was warmed to room temperature and stirred for 40 minutes, after which it was cooled again to 0 °C. 10.0 ml (109 mmol, 1.5 equiv.) (*N*,*N*)-dimethylcarbamoyl chloride were added and the reaction stirred for 21 hours at room temperature, after which it was carefully diluted with 100 ml of water. The organic components were extracted with EtOAc (3x100 ml), the combined phases washed once with saturated, aqueous NaCl-solution and a 10%, aqueous LiCl-solution, after which they were dried over Na_2SO_4 and concentrated under reduced pressure. The crude material was then crystallized from acetone (*approx.* 150 ml) and 20.8 g (65.5 mmol, **88%** (2 steps)) carbamate collected as a white powder

R_f = 0.46 (PE/ EtOAc = 1:1 (v/v));

m.p. = 117 - 119 °C (acetone);

HR-ESI-MS: $[C_{12}H_{16}O_4NBrNa]^+$: calc. m/z = 340.0160, found m/z = 340.0150;

^{1}H-NMR (400 MHz; $CDCl_3$): δ [ppm] = 6.60 (d, *J* = 2.8 Hz, **1H**), 6.45 (d, *J* = 2.8 Hz, **1H**), 5.18 (s, **2H**), 3.88 (s, **3H**), 3.81 (s, **3H**), 2.96 (s, **6H**);

^{13}C-NMR (101 MHz; $CDCl_3$): δ [ppm] = 159.8 (**1C**), 156.8 (**1C**), 156.3 (**1C**), 138.3 (**1C**), 106.1 (**1C**), 103.4 (**1C**), 99.1 (**1C**), 66.9 (**1C**), 56.5 (**1C**), 55.7 (**1C**), 36.7 (**1C**), 36.1 (**1C**).

---

[10] The synthesis was carried out by following a protocol described by Wright et al. with the recorded spectrometric and spectroscopic data being in accordance with those previously reported by the authors.[14]

lactonization (synthesis of **17**): The previously prepared carbamate (20.8 g ,65.5mmol, 1.0 equiv.) was dissolved in 314 ml dry THF in a flame-dried one-neck 500-ml round-bottom flask under an inert atmosphere. The solution was then cooled to –78 °C and treated with a ^{n}BuLi-solution (1.6M in hexanes; 74.7 ml, 119 mmol, 3.8 equiv.). After stirring the reaction at –78 °C for 45 minutes, the cooling bath was removed and the stirring continued for additional 135 minutes at room temperature. Upon completion (TLC), any unreacted Li-base residues were carefully neutralized by the addition of wet MeOH (carefully added until no more gas-formation was observed) and 150 ml of water, and the volatiles removed under reduced pressure. The cloudy aqueous solution was extracted with copious amounts of EtOAc (4x200 ml) and the organic phases dried over Na_2SO_4. After concentration *in vacuo*, the obtained solid residue was crystallized from acetone (200 ml) to yield phthalide **17** (3.20 g, 15.1 mmol, **48%**) as a white powder.

$\mathbf{R_f}$ = 0.66 (EtOAc);

m.p. = 150 - 152 °C (acetone);

HR-ESI-MS: $[C_{10}H_{10}O_4Na]^+$: calc. m/z = 217.0477, found m/z = 217.0478;

^{1}H-NMR (400 MHz; $CDCl_3$): δ [ppm] = 6.48 (s, **1H**), 6.42 (d, *J* = 1.8 Hz, **1H**), 5.17 (s, **2H**), 3.95 (s, **3H**), 3.89 (s, **3H**);

^{13}C-NMR (101 MHz; $CDCl_3$): δ [ppm] = 169.1 (**1C**), 166.9 (**1C**), 159.9 (**1C**), 151.8 (**1C**), 106.8 (**1C**), 99.0 (**1C**), 97.7 (**1C**), 68.7 (**1C**), 56.1 (**2C**). [11]

[11] The recorded spectrometric and spectroscopic data are in accordance with those reported by Ward et al.[15]

2.3 Synthesis of tetracycline precursor **9**

[4+2]-cycloaddition product **19**

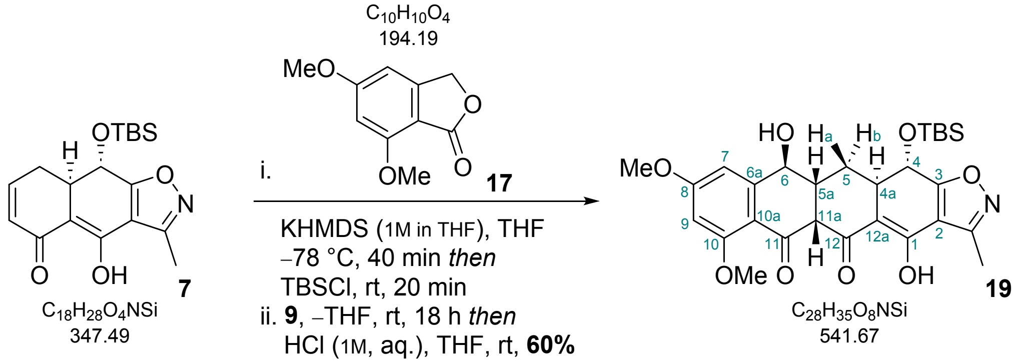

Phthalide **17** (0.46 g, 2.37 mmol, 1.5 equiv.) was dissolved in 4.7 ml dry THF in a flame-dried 10-ml one-neck round-bottom flask and the formed suspension cooled to –78 °C. A 1M (THF) solution of KHMDS-solution (3.4 ml, 3.36 mmol, 2.1 equiv.) was added and the cloudy, red-colored solution stirred for 40 minutes under an inert atmosphere. TBSCl (0.55 g, 3.64 mmol, 2.3 equiv.) was then added and the reaction warmed to room temperature over a period of 20 minutes after which 0.55 g (1.58 mmol, 1.0 equiv.) of decalin **7** were added and the solvent-amount reduced under vacuum to 10% of its original volume. At this point, it is mandatory to highlight the importance of ensuring a concentrated reaction medium while performing the cycloaddition step. Furthermore, the complete removal of the solvent (THF) from the deprotonation step prior to cycloaddition has represented a drawback, with the intended transformation failing to set in. Enone **7** must be therefore added as a concentrated ethereal solution (THF) to the reaction matrix and the solvent removal step performed afterwards. The concentrated, dark-green-colored slurry was then stirred for additional 18 hours. Upon completion, the reaction was diluted with 10 ml of water and 10 ml MTBE. The organic components were then extracted with MTBE (3x20 ml) and the combined organic phases dried over Na_2SO_4. Upon removal of the volatiles under reduced pressure, the residue was quickly (!) purified *via* flash column chromatography (SiO_2, PE/ EtOAc = 3:2 (v/v) with 1.5 vol-% NEt_3). The fractions containing the impure cycloaddition product (**R_f** = 0.89 (PE/ EtOAc = 7:3 (v/v))) were then collected and concentrated under reduced pressure.[12] The oily residue was redissolved in 5 ml THF and the solution acidified by the addition of five drops of HCl-solution (1M, aq.). The mixture was then transferred to a separatory funnel, diluted with water and extracted with EtOAc (4x80 ml). The combined phases were then dried over Na_2SO_4, the volatiles removed under reduced pressure and the crude product purified *via* preparatory RP-HPLC (injection volume: 1.0 ml (MeCN), elution with H_2O/ MeCN-gradient, flow-rate; 15.00 ml/ min). The fractions eluting at 44-45 min (elution with pure MeCN) were collected and concentrated to give precursor **19** (0.52 g, 0.95 mmol, **60%**) as a bright yellow solid.

R_f = 0.33 (PE/ EtOAc = 2:3 (v/v));

m.p. = 130 - 133 °C;

$[\alpha]_D^{22.1}$ = +175.58° ($CHCl_3$, *7.92*);

HR-ESI-MS: $[C_{28}H_{35}O_8NSiNa]^+$: calc. m/z = 564.2030, found m/z = 564.2034;

[12] In spite of recent accounts,[16] the isolation and characterization of cycloaddition product was not attempted due to stability concerns. Therefore, in order to maintain the integrity of the newly constructed architecture, the isolated product was quickly converted to product **19** under the action of dilute acid.

^{1}**H-NMR** (400 MHz; $CDCl_3$): δ [ppm] = 6.51 (s, **1H**), 6.42 (d, *J* = 2.0 Hz, **1H**), 4.96 (d, *J* = 7.1 Hz, **1H**), 4.38 (s, **1H**), 3.89 (s, **3H**), 3.84 (s, **3H**), 3.80 (d, *J* = 5.2 Hz, **1H**), 2.93–2.95 (m, **2H**), 2.36 (br. s, **1H**), 2.48 (s, **3H**), 2.08–2.11 (m, **2H**), 0.92 (s, **9H**), 0.21 (s, **3H**), 0.15 (s, **3H**);

^{13}**C-NMR** (101 MHz; $CDCl_3$): δ [ppm] = 187.7 (**1C**), 185.5 (**1C**), 179.1 (**1C**), 178.1 (**1C**), 164.8 (**1C**), 162.6 (**1C**), 157.8 (**1C**), 146.7 (**1C**), 113.9 (**1C**), 111.9 (**1C**), 105.7 (**1C**), 102.2 (**1C**), 99.3 (**1C**), 72.1 (**1C**), 64.8 (**1C**), 56.1 (**1C**), 55.7 (**1C**), 54.3 (**1C**), 43.0 (**1C**), 34.8 (**1C**), 25.7 (**3C**), 24.7 (**1C**), 18.2 (**1C**), 10.7 (**1C**), –4.5 (**1C**), –4.8 (**1C**).

Elimination-product **20**

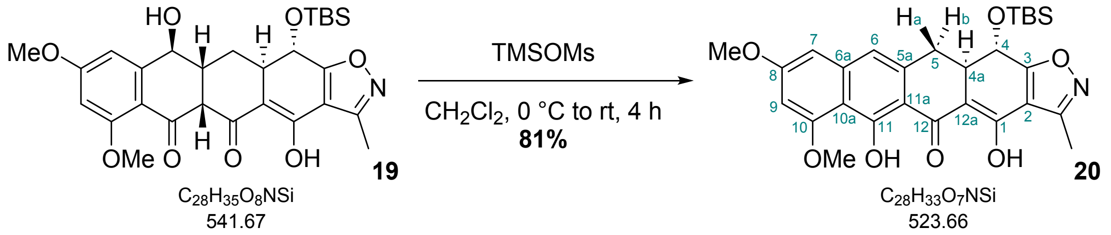

Precursor **19** (0.52 g, 0.95 mmol, 1.0 equiv.) was dissolved in 3.8 ml dry CH_2Cl_2 in a flame-dried 10-ml one-neck round-bottom flask and the formed solution cooled to 0 °C. TMSOMs (0.7 ml, 4.23 mmol, 4.5 equiv.) was then added and the red-colored solution stirred for 4 hours at room temperature under an inert atmosphere. Upon completion, the mixture was treated with 3 ml saturated, aqueous $NaHCO_3$-solution and diluted with water and EtOAc. Extraction with EtOAc (6x150 ml), followed by drying of the combined phases over Na_2SO_4 and removal of the volatiles under reduced pressure afforded the crude reaction product. Purification *via* flash column chromatography (SiO_2, PE/ EtOAc = 4:1→3:2 (v/v)) gave the aimed tetracycline precursor **20** (0.40 g, 0.77 mmol, **81%**) as a bright-yellow solid.

$\mathbf{R_f}$ = 0.78 (PE/ EtOAc = 3:2 (v/v));

m.p. = decomp. >120 °C;

$[\alpha]_D^{21.7}$ = +294.44° ($CHCl_3$, *3.00*);

HR-ESI-MS: $[C_{28}H_{33}NO_7SiNa]^+$: calc. m/z = 546.1924, found m/z = 546.1921;

^{1}**H-NMR** (400 MHz; $CDCl_3$): δ [ppm] = 6.91 (s, **1H**), 6.60 (d, *J* = 2.3 Hz, **1H**), 6.44 (d, *J* = 2.2 Hz, **1H**), 4.88 (d, *J* = 12.0 Hz, **1H**), 3.90 (s, **3H**), 3.89 (s, **3H**), 3.27 (dd, *J* = 14.3, 4.9 Hz, **1H**), 3.19 (td, *J* = 12.7, 12.6, 4.9 Hz, **1H**), 2.73 (t, *J* = 13.3, 13.3 Hz, **1H**), 2.50 (s, **3H**), 1.03 (s, **9H**), 0.31 (s, **3H**), 0.24 (s, **3H**);

^{13}**C-NMR** (101 MHz; $CDCl_3$): δ [ppm] = 190.1 (**1C**), 175.7 (**1C**), 168.5 (**1C**), 164.2 (**1C**), 161.9 (**1C**), 161.0 (**1C**), 157.0 (**1C**), 141.5 (**1C**), 136.3 (**1C**), 116.5 (**1C**), 111.0 (**1C**), 110.7 (**1C**), 109.3 (**1C**), 102.7 (**1C**), 99.1 (**1C**), 98.2 (**1C**), 71.8 (**1C**), 56.3 (**1C**), 55.6 (**1C**), 41.4 (**1C**), 33.9 (**1C**), 25.9 (**3C**), 18.4 (**1C**), 10.9 (**1C**), –4.2 (**1C**), –4.5 (**1C**).

Ketol **8**

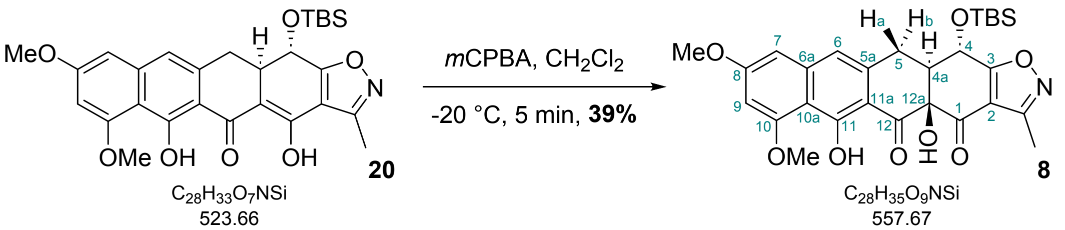

73.5 mg (0.14 mmol, 1.0 equiv.) **20** were dissolved in 2.8 ml dry CH_2Cl_2 in a flame-dried 10-ml one-neck round-bottom flask and the formed suspension cooled to –20 °C. *m*CPBA (77 wt%, tech. grade; 34.6 mg, 0.15 mmol, 1.1 equiv.) was then added at once and the red-colored solution stirred for **five minutes (!)** under an inert atmosphere. The reaction was then diluted with 2 ml of water and the pH adjusted to 5 by the addition of saturated, aqueous $NaHCO_3$-solution. The aqueous phase was then extracted with MTBE (3x15 ml)[13], the combined organic phases dried over Na_2SO_4 and the volatiles removed under reduced pressure. The thus obtained crude product was purified *via* preparatory RP-HPLC (injection volume: 1.0 ml (MeCN), elution with H_2O/ MeCN-gradient, flow-rate; 15.00 ml/ min). The fractions eluting at 50-52 min (elution with 20% H_2O, 80% MeCN) were collected and concentrated to give the aimed ketol **8** (29.5 mg, 54.7 µmol, **39%**) as a yellow-colored solid.

$\mathbf{R_f}$ = 0.56 (PE/ EtOAc = 3:2 (v/v));

m.p. = 197 - 198 °C;

$[\alpha]_D^{29.5}$ = +269.11° (CHCl3, *4.10*);

HR-ESI-MS: $[C_{28}H_{33}NO_8SiNa]^+$: calc. m/z = 562.1873, found m/z = 562.1883;

^{1}H-NMR (400 MHz; $CDCl_3$): δ [ppm] = 13.80 (s, **1H**), 6.87 (s, **1H**), 6.57 (d, *J* = 2.2 Hz, **1H**), 6.41 (d, *J* = 2.2 Hz, **1H**), 5.21 (d, *J* = 9.0 Hz, **1H**), 4.17 (br. s, **1H**), 3.93(s, **3H**), 3.90 (s, **3H**), 3.18–3.20 (m, **2H**), 2.74 (td, *J* = 9.1, 9.1, 7.1 Hz, **1H**), 2.45 (s, **3H**), 1.02 (s, **9H**), 0.35 (s, **3H**), 0.32 (s, **3H**);

^{13}C-NMR (101 MHz; $CDCl_3$): δ [ppm] = 196.4 (**1C**), 187.9 (**1C**), 179.0 (**1C**), 166.5 (**1C**), 162.8 (**1C**), 161.3 (**1C**), 158.5 (**1C**), 142.3 (**1C**), 136.8 (**1C**), 116.3 (**1C**), 112.6 (**1C**), 110.4 (**1C**), 110.1 (**1C**), 99.4 (**1C**), 98.3 (**1C**), 76.3 (**1C**), 66.2 (**1C**), 56.3 (**1C**), 55.6 (**1C**), 49.8 (**1C**), 27.0 (**1C**), 25.9 (**3C**), 18.4 (**1C**), 10.7 (**1C**), –4.3 (**1C**), –4.6 (**1C**).

[13] *The extraction with MTBE was typically carried out until the otherwise orange-colored organic phase turned colorless. A red coloration of the aqueous phase is to be expected.*

Rearranged ketol 9

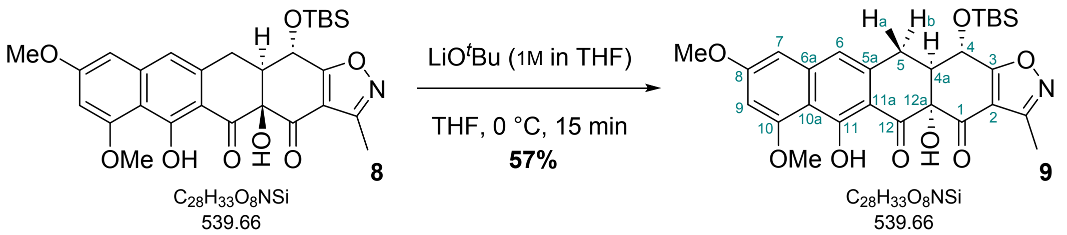

Ketol **8** (29.5 mg, 54.7 µmol, 1.0 equiv.) was dissolved in 0.7 ml dry THF in a flame-dried 5-ml one-neck round-bottom flask at 0 °C and the solution treated with 73.8 µl (73.8 µmol, 1.4 equiv.) LiO^{t}Bu-solution (1M in THF). The formed brown solution was then stirred at 0 °C for 15 minutes after which 4 ml of water were added. The pH was adjusted to 5 by the addition of 1M aqueous HCl-solution and the aqueous phase extracted with MTBE (3x10 ml)[14]. The combined organic phases were dried over Na_2SO_4, the volatiles removed under reduced pressure and the crude product purified *via* preparatory RP-HPLC (injection volume: 1.0 ml (MeCN), elution with H_2O/ MeCN-gradient, flow-rate; 15.00 ml/ min). The fractions eluting at 45-57 min (elution with 20% H_2O, 80% MeCN) were collected and concentrated to give the rearranged ketol **9** (16.8 mg, 31.1 µmol, **57%**) as a yellow solid.

$\mathbf{R_f}$ = 0.78 (PE/ EtOAc = 3:2 (v/v));

m.p. = 145 - 147 °C;

$[\alpha]_D^{27.3}$ = +106.67° ($CHCl_3$, *1.00*);

HR-ESI-MS: $[C_{28}H_{33}NO_8SiNa]^+$: calc. m/z = 562.1873, found m/z = 562.1872;

^{1}**H-NMR** (400 MHz; $CDCl_3$): δ [ppm] = 6.89 (s, **1H**), 6.58 (d, *J* = 2.3 Hz, **1H**), 6.45 (d, *J* = 2.2 Hz, **1H**), 4.84 (d, *J* = 9.1 Hz, **1H**), 4.72 (br. s, **1H**), 3.96(s, **3H**), 3.93 (s, **3H**), 3.62 (ddd, *J* = 16.8, 4.4, 1.7 Hz, **1H**,), 3.16 (dd, *J* = 17.1, 2.7 Hz, **1H**), 2.92 (ddd, *J* = 9.1, 4.3, 2.7 Hz, **1H**), 2.55 (s, **3H**), 0.95 (s, **9H**), 0.16 (s, **3H**), 0.06 (s, **3H**);

^{13}**C-NMR** (101 MHz; $CDCl_3$): δ [ppm] = 194.9 (**1C**), 189.3 (**1C**), 179.9 (**1C**), 167.9 (**1C**), 163.2 (**1C**), 161.7 (**1C**), 157.8 (**1C**), 142.7 (**1C**), 135.1 (**1C**), 118.1 (**1C**), 113.4 (**1C**), 110.4 (**1C**), 108.9 (**1C**), 99.5 (**1C**), 98.5 (**1C**), 79.8 (**1C**), 56.3 (**1C**), 65.0 (**1C**), 55.7 (**1C**), 50.9 (**1C**), 26.3 (**1C**), 25.8 (**3C**), 18.2 (**1C**), 10.8 (**1C**), –4.7 (**1C**), –4.7 (**1C**).

[14] *The extraction with MTBE was typically carried out until the otherwise yellow-colored organic phase turned colorless. A brown coloration of the aqueous phase is to be expected.*

4 References

[1] Barco, A.; Benetti, S.; De Risi, C.; Marchetti, P.; Pollini, G.; Zanirato, V. D-(−)-Quinic acid: a chiron store for natural product synthesis. *Tetrahedron: Asymmetry.* **1997**, *8* (21), 3515–3545. DOI: 10.1016/S0957-4166(97)00471-0

[2] Kawashima, H.; Sakai, M.; Kaneko, Y.; Kobayashi, Y. Further study on synthesis of the cyclobakuchiols. *Tetrahedron.* **2015**, *71* (16), 2387–2392. DOI: 10.1016/j.tet.2015.02.09

[3] Trost, B.; Romero, A. Synthesis of optically active isoquinuclidines utilizing a diastereoselectivity control element. *J. Org. Chem.* **1986**, *51* (12), 2332–2342. DOI: 10.1021/jo00362a030

[4] Wang, Z.; Miller, S.; Anderson, O.; Shi, Y. A Class of *C*2 and Pseudo *C*2 Symmetric Ketone Catalysts for Asymmetric Epoxidation. Conformational Effect on Catalysis. *J. Org. Chem.* **1999,** *64* (17), 6443–6458. DOI: 10.1021/jo9908849

[5] Böse, D.; Frey, W.; Pietruszka, J. The 'Mikami'-Catalyst in Enantioselective Diels–Alder Reactions of Juglone-Based Dienophiles with Different 1-Oxygenated Dienes: An Investigation on the Substitution Pattern Dependent Regioselectivity. *Synthesis.* **2014**, *46* (18), 2524–2532. DOI: 10.1055/s-0034-1378230

[6] Hylse, O.; Maier, L.; Kučera, R.; Perečko, T.; Svobodová, A.; Kubala, L.; Paruch, K.; Švenda, J. A Concise Synthesis of Forskolin. *Angew. Chem. Int. Ed.* **2017**, *56* (41), 12586–12589. DOI: 10.1002/anie.201706809

[7] Kim, T.; Lee, B.; Lee, H.; Chung, K.; Kim, J. Synthesis and Characterization of Bisimidazolylfuroxan Derivatives. *Bull. Korean Chem. Soc.* **2013**, *34* (6), 1864–1866. DOI: 10.5012/bkcs.2013.34.6.1864

[8] Adembri, G.; Giorgi, G.; Lampariello, R.; Paoli, M.; Sega, A. Chemistry of 4-Hydroxy-2-cyclopentenone Derivatives. *J. Chem. Soc. Perkin Trans. 1*, **2000**, *44*, 2649–2656. DOI: 10.1002/ejoc.201000704

[9] Takikawa, H.; Ishikawa, Y.; Yoshinaga, Y.; Hashimoto, Y.; Kusumi, T.; Suzuki, K. Pleospdione, A Tricyclic Natural Product with Dense Oxygenation at the A-Ring: Total Synthesis and Incongruity of the Originally Assigned Structure and its C3-Epimer. *Bull. Chem. Soc. Jpn.* **2016**, *89* (8), 941–954. DOI: 10.1246/bcsj.20160134

[10] Armarego, W.; Chai, C. *Purification of Laboratory Chemicals*, 6th edition; Butterworth-Heinemann, 2009.

[11] Fulmer, G.; Miller, A.; Sherden, N.; Gottlieb, H.; Nudelman, A.; Stoltz, B.; Bercaw, J.; Goldberg, K. NMR Chemical Shifts of Trace Impurities: Common Laboratory Solvents, Organics, and Gases in Deuterated Solvents Relevant to the Organometallic Chemist. *Organometallics.* **2010**, *29*, 2176–2179. DOI: 10.1021/om100106e

[12] Kawashima, H.; Sakai, M.; Kaneko, Y.; Kobayashi, Y. Further study on synthesis of the cyclobakuchiols. *Tetrahedron.* **2015**, *71* (16), 2387–2392. DOI: doi.org/10.1016/j.tet.2015.02.092

[13] Das, O.; Paria, S.; Paine, T. Copper(II)-mediated oxidation of 1,2-dioxime to furoxan. *Tetrahedron Lett.* **2008**, *49* (41), 5924–5927. DOI: 10.1016/j.tetlet.2008.07.148

[14] Wright, N.; ElSohly, A.; Snyder, S. Syntheses of Cyclotriveratrylene Analogues and Their Long Elusive Triketone Congeners. *Org. Lett.* **2014**, *16* (14), 3644–3647. DOI: 10.1021/ol501284s

[15] Clarke, S.; Kasurn, B.; Prager, R.; Ward, D. Central nervous system active compounds. XII. The synthesis and properties of 3-dimethylaminomethylene phthalides. *Aust. J. Chem*. **1983**, *36* (12), 2483–2491. DOI: 10.1071/CH9832483

[16] Schneider, F.; Samarin, K.; Zanella, S.; Gaich, T. Total synthesis of the complex taxane diterpene canataxpropellane. *Science.* **2020**, *367*, 676–681. DOI: 10.1126/science.aay9173

5 Spectra and crystallographic parameters

4.1 Spectra

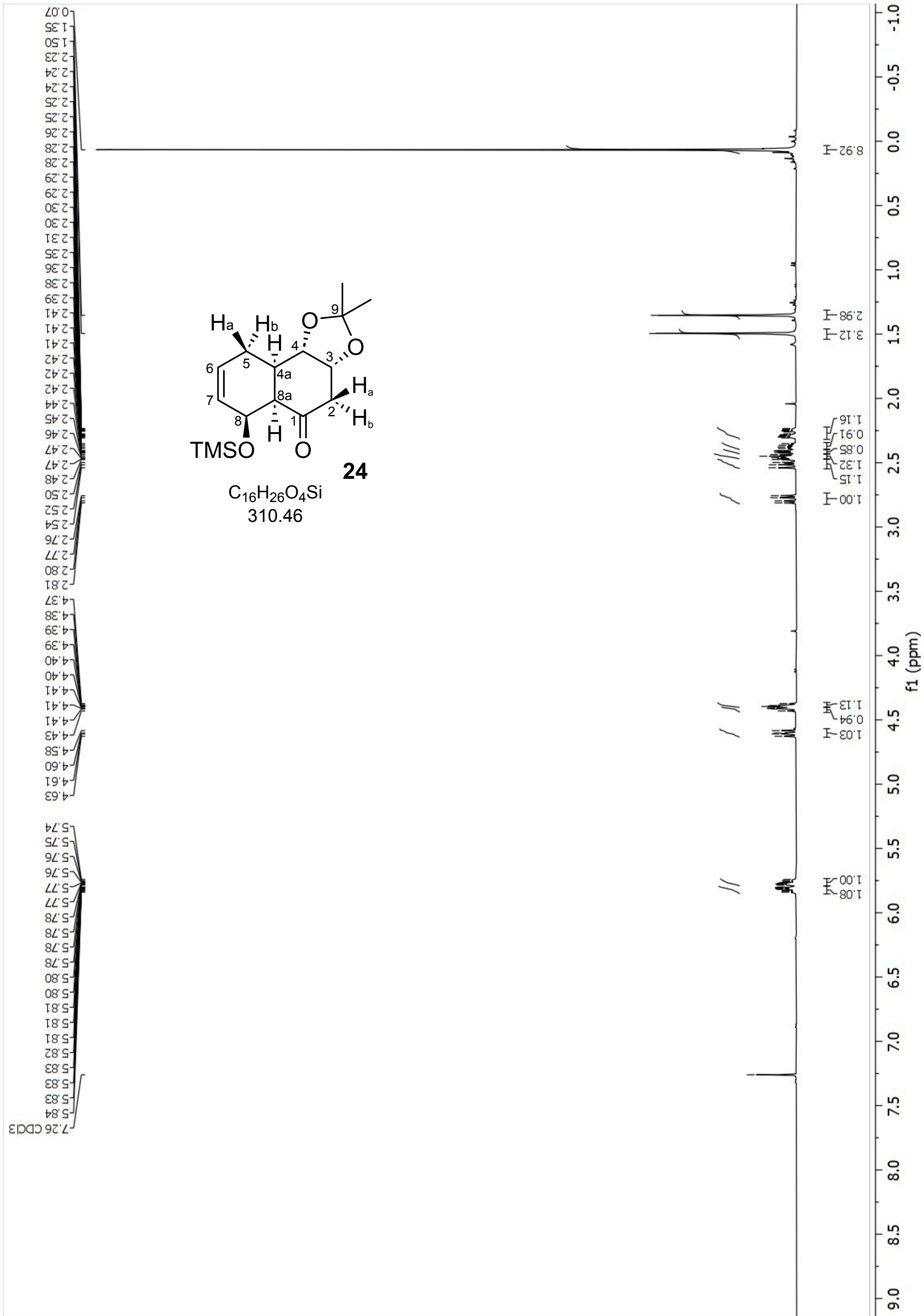

Spectrum 1 ^{1}H-NMR spectrum of compound **24** measured in $CDCl_3$ at 400 MHz.

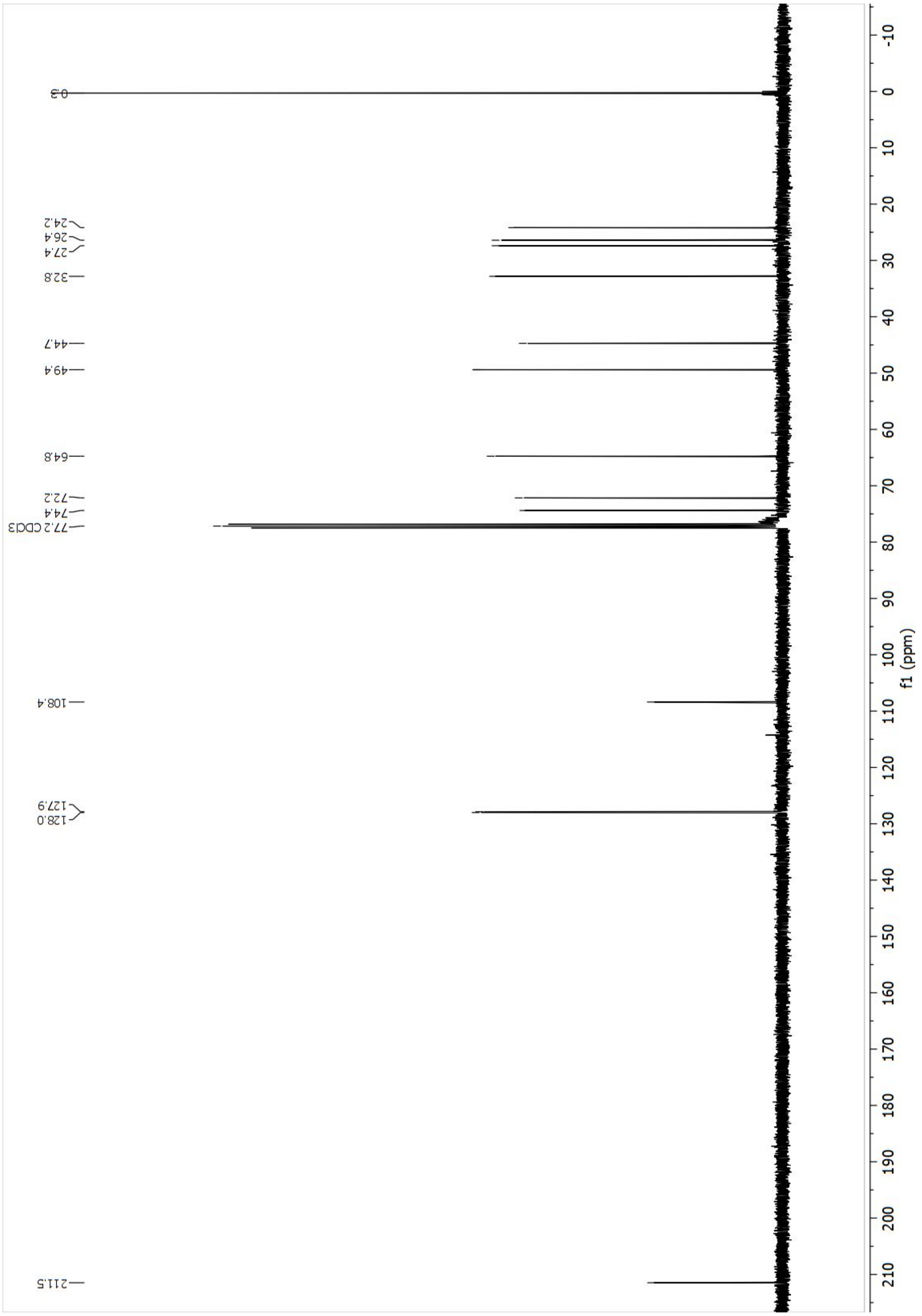

Spectrum 2 ^{13}C-NMR spectrum of compound **24** measured in $CDCl_3$ at 101 MHz.

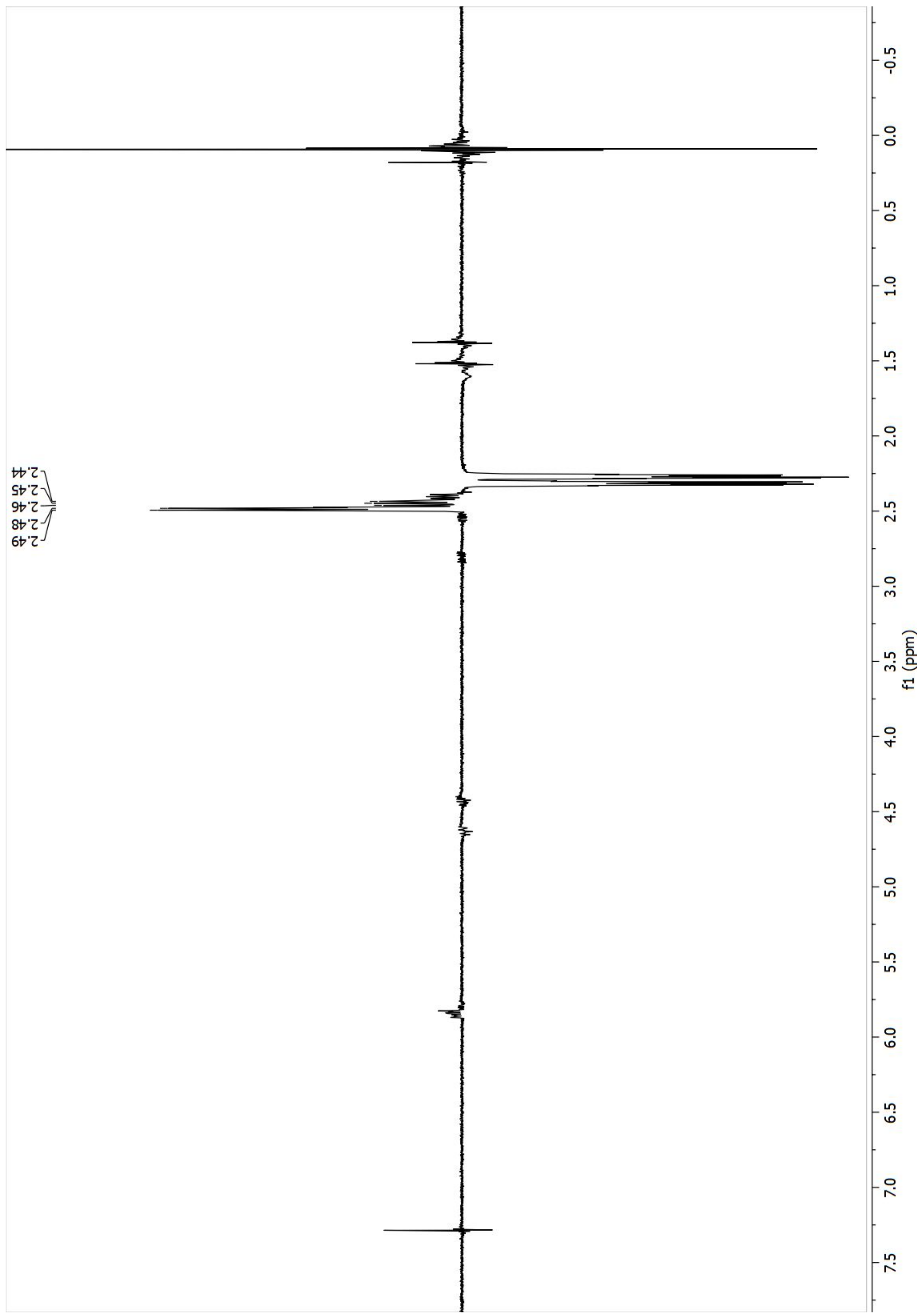

Spectrum 3 Nuclear Overhauser effects with excitation of the signal at 2.27 ppm measured in $CDCl_3$ at 400 MHz.

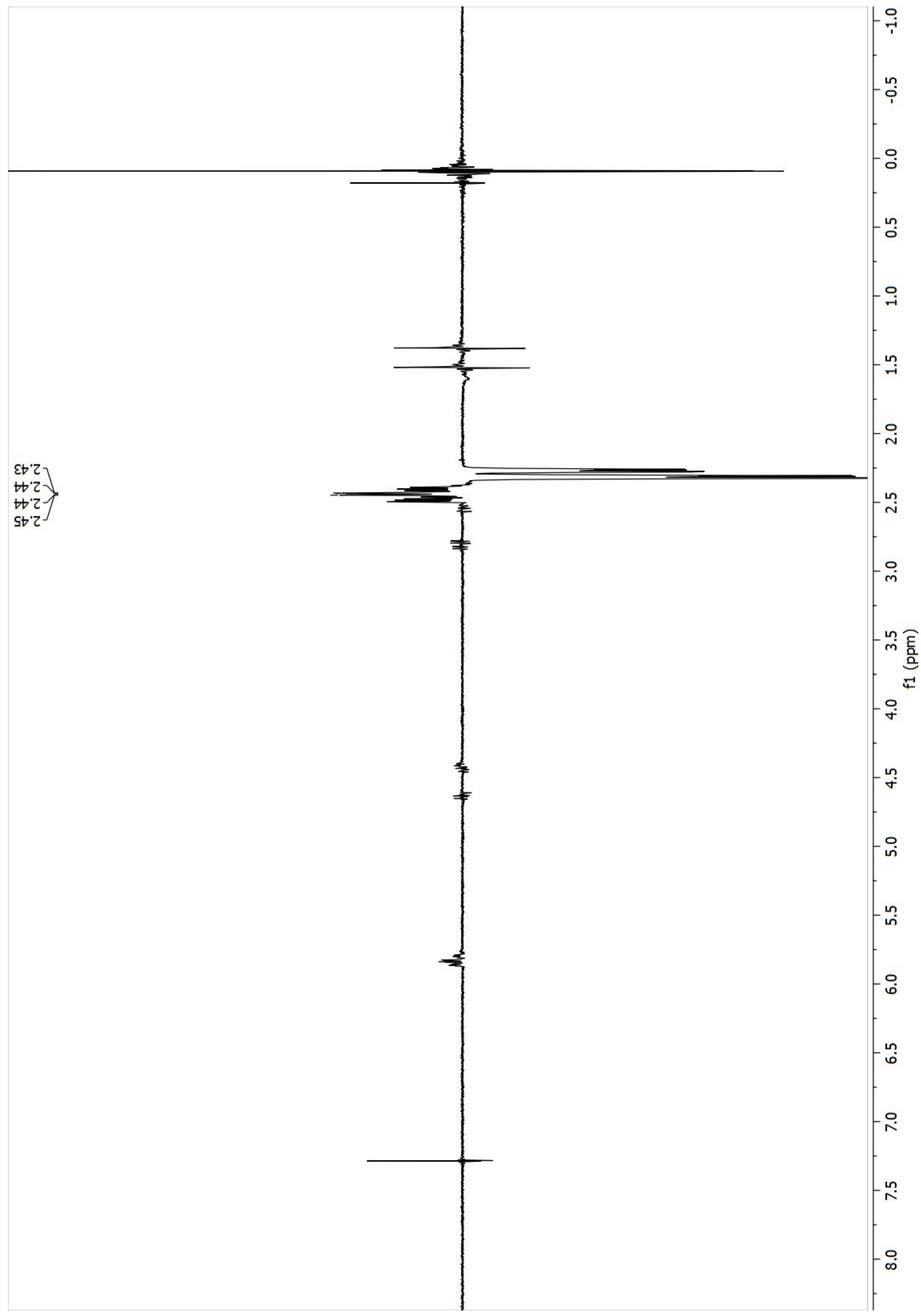

Spectrum 4 Nuclear Overhauser effects with excitation of the signal at 2.37 ppm measured in $CDCl_3$ at 400 MHz.

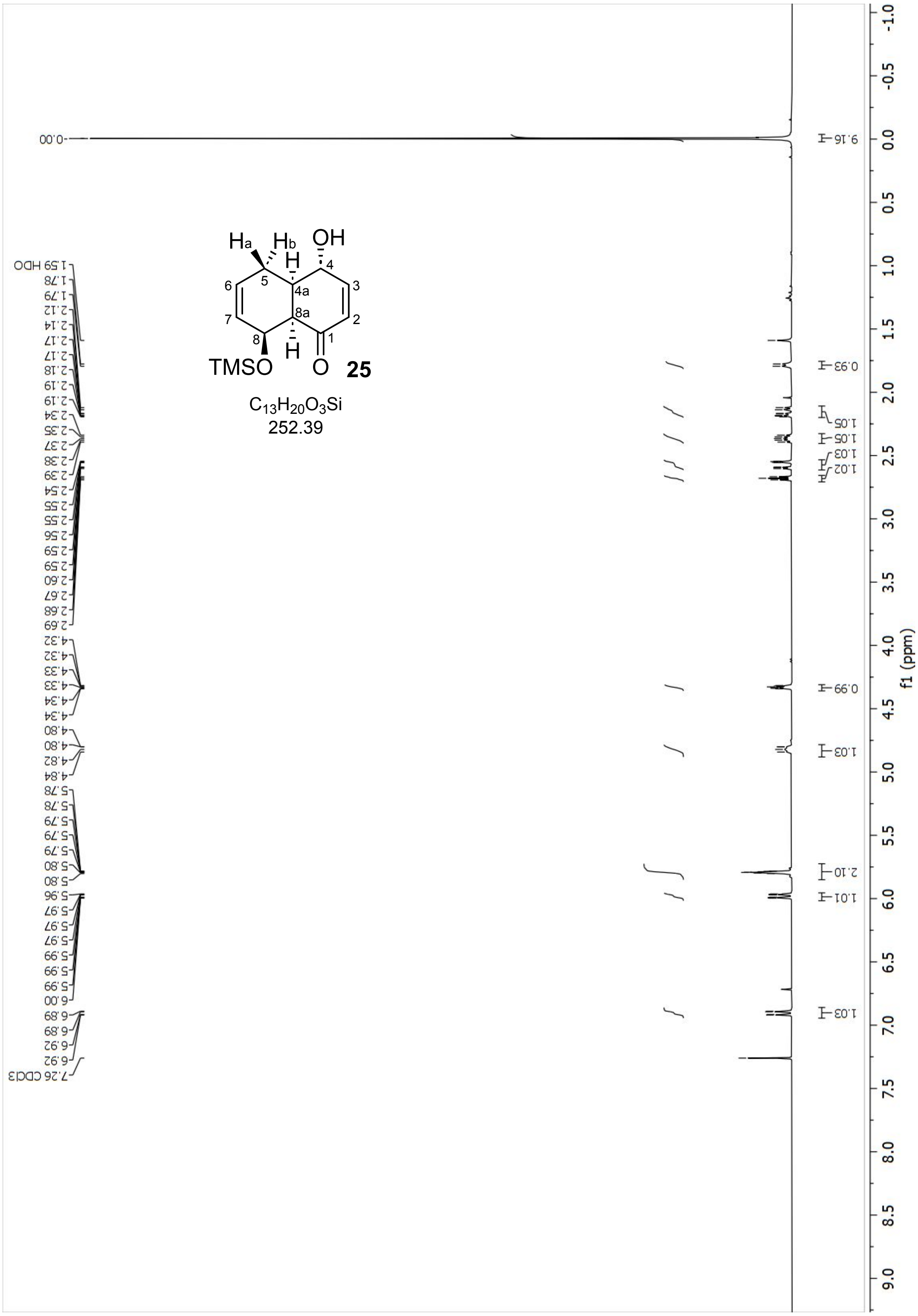

Spectrum 5 ^{1}H-NMR spectrum of compound **25** measured in $CDCl_3$ at 400 MHz.

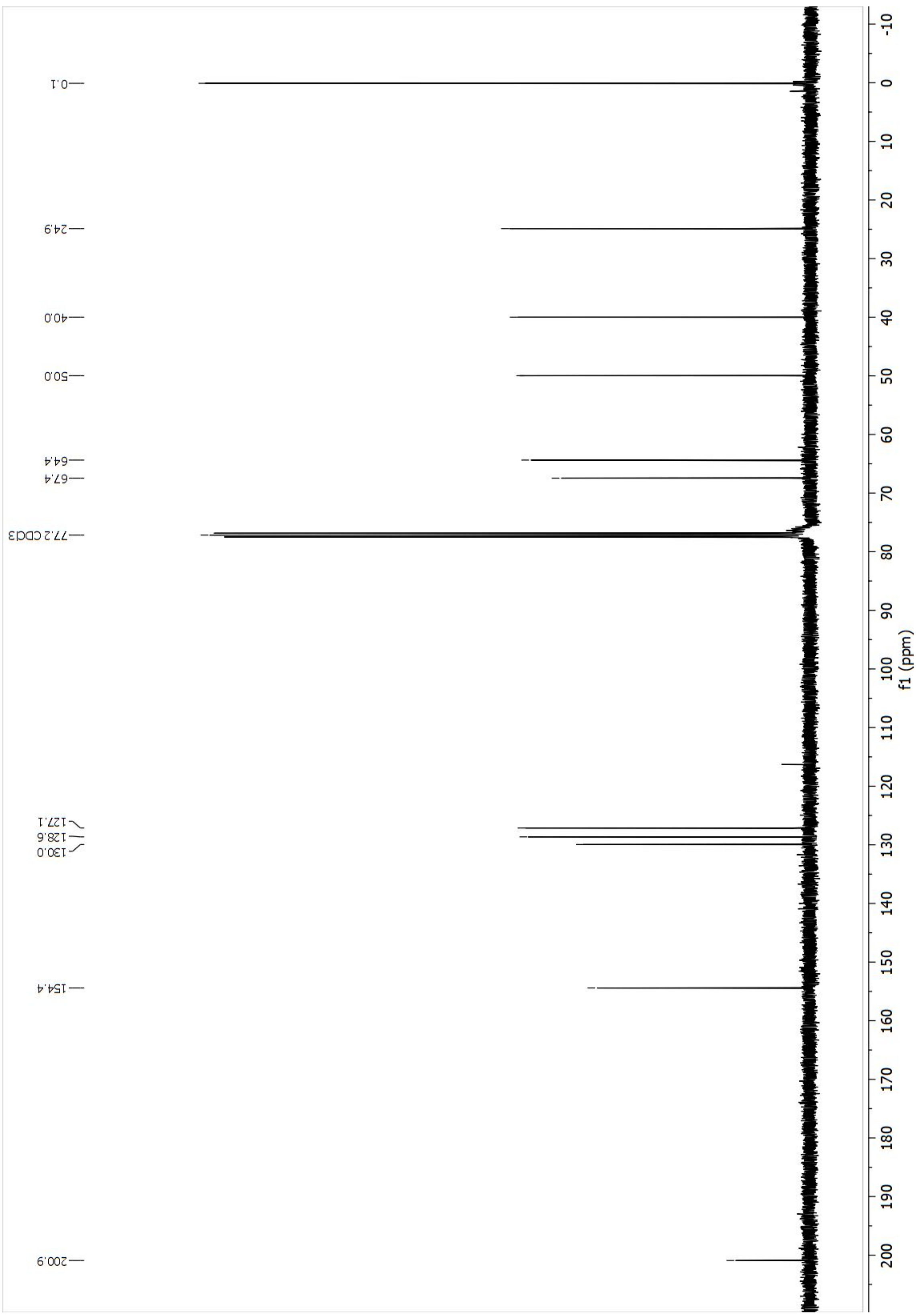

Spectrum 6 ^{13}C-NMR spectrum of compound **25** measured in $CDCl_3$ at 101 MHz.

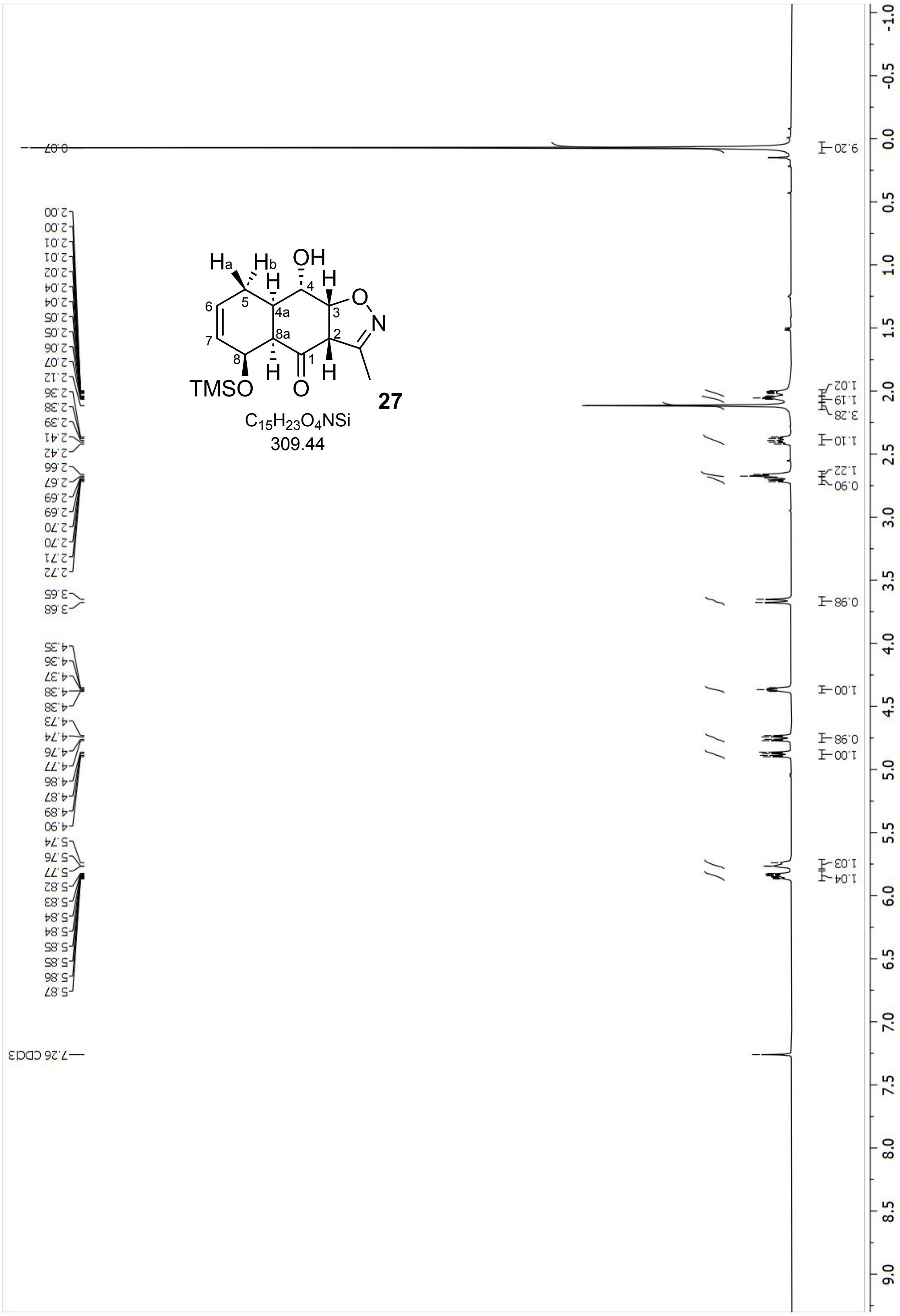

Spectrum 7 ^{1}H-NMR spectrum of compound **27** measured in $CDCl_3$ at 400 MHz.

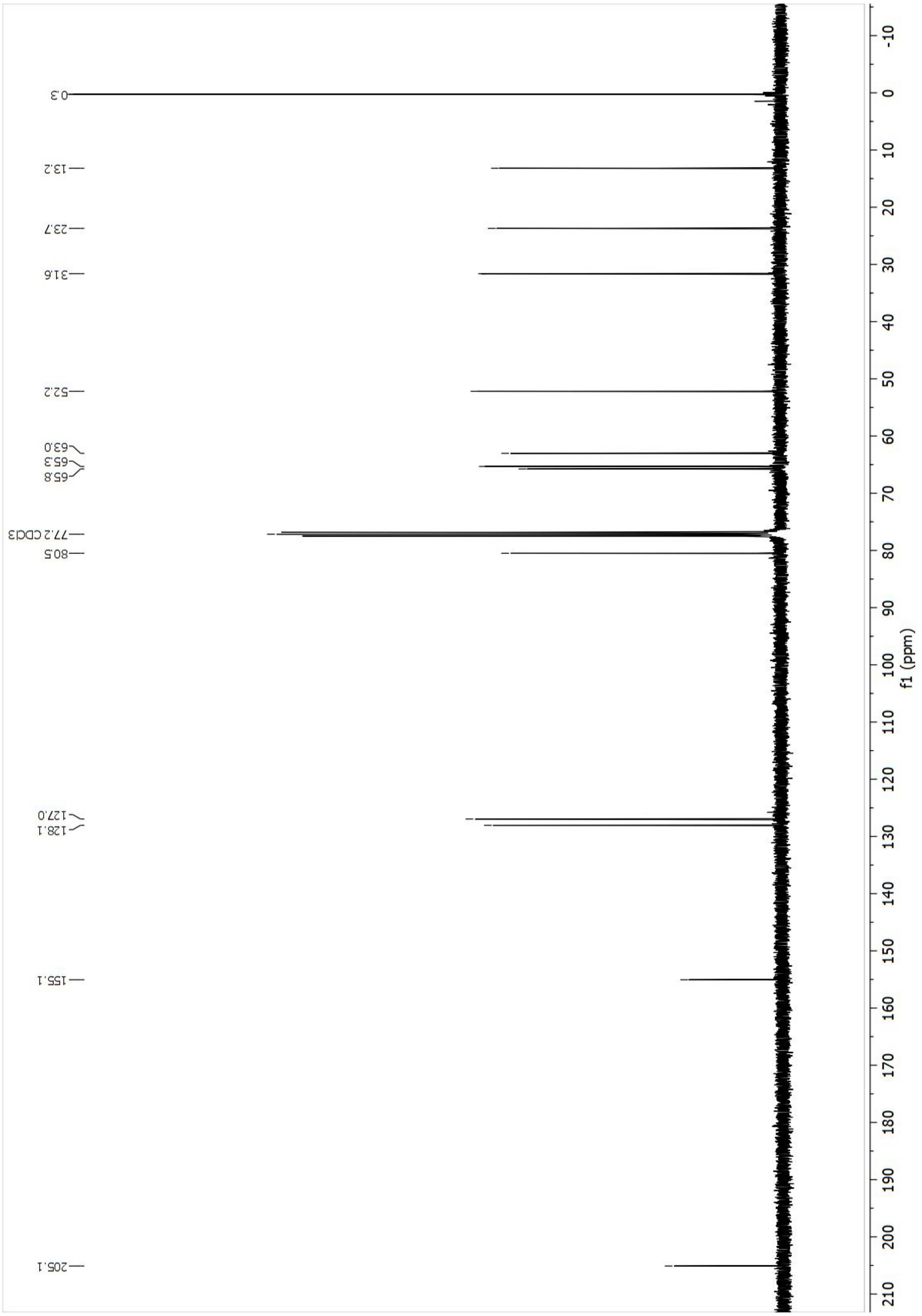

Spectrum 8 ^{13}C-NMR spectrum of compound **27** measured in $CDCl_3$ at 101 MHz.

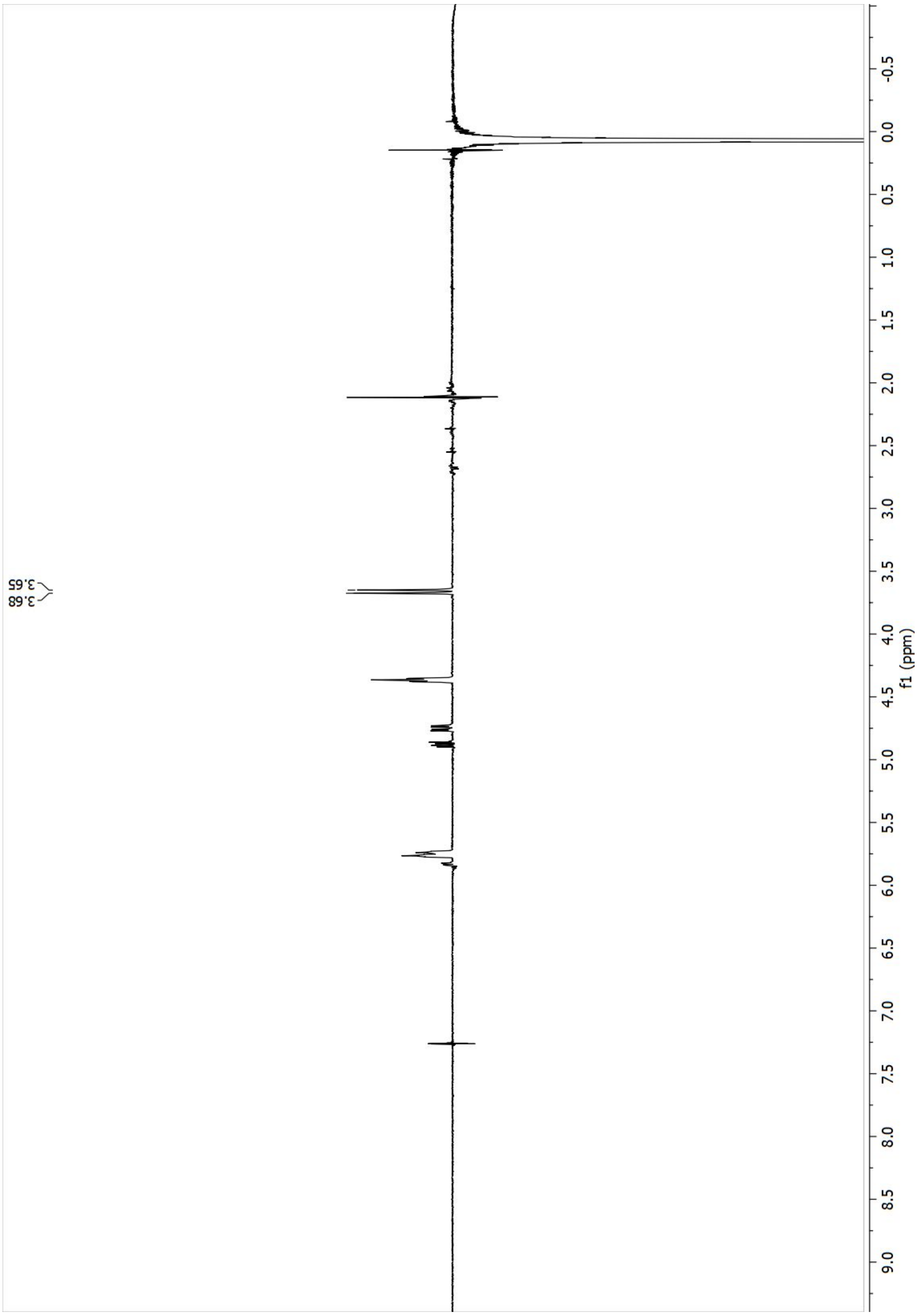

Spectrum 9 Nuclear Overhauser effects with excitation of the signal at 0.09 ppm measured in $CDCl_3$ at 400 MHz.

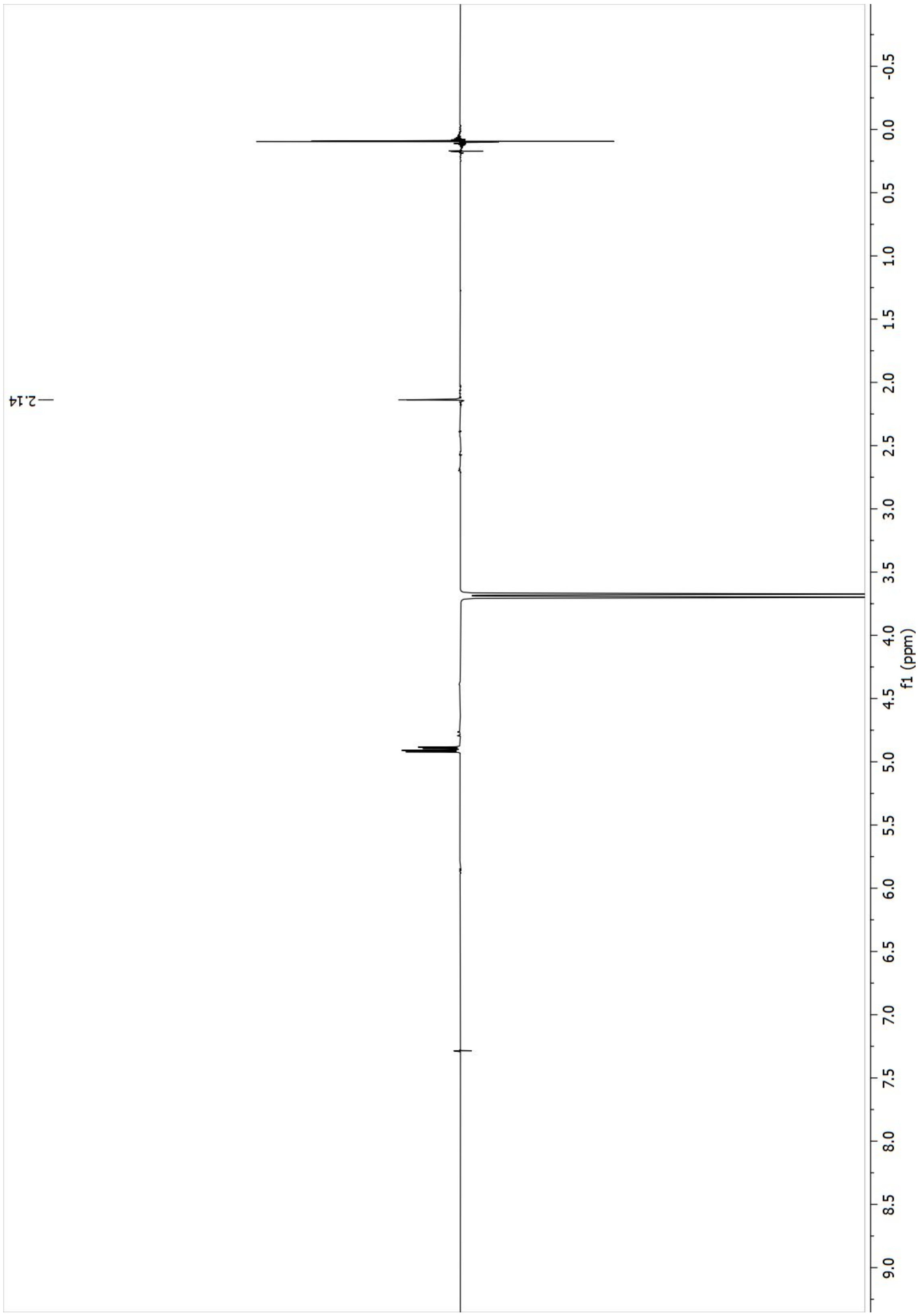

Spectrum 10 Nuclear Overhauser effects with excitation of the signal at 3.69 ppm measured in $CDCl_3$ at 400 MHz.

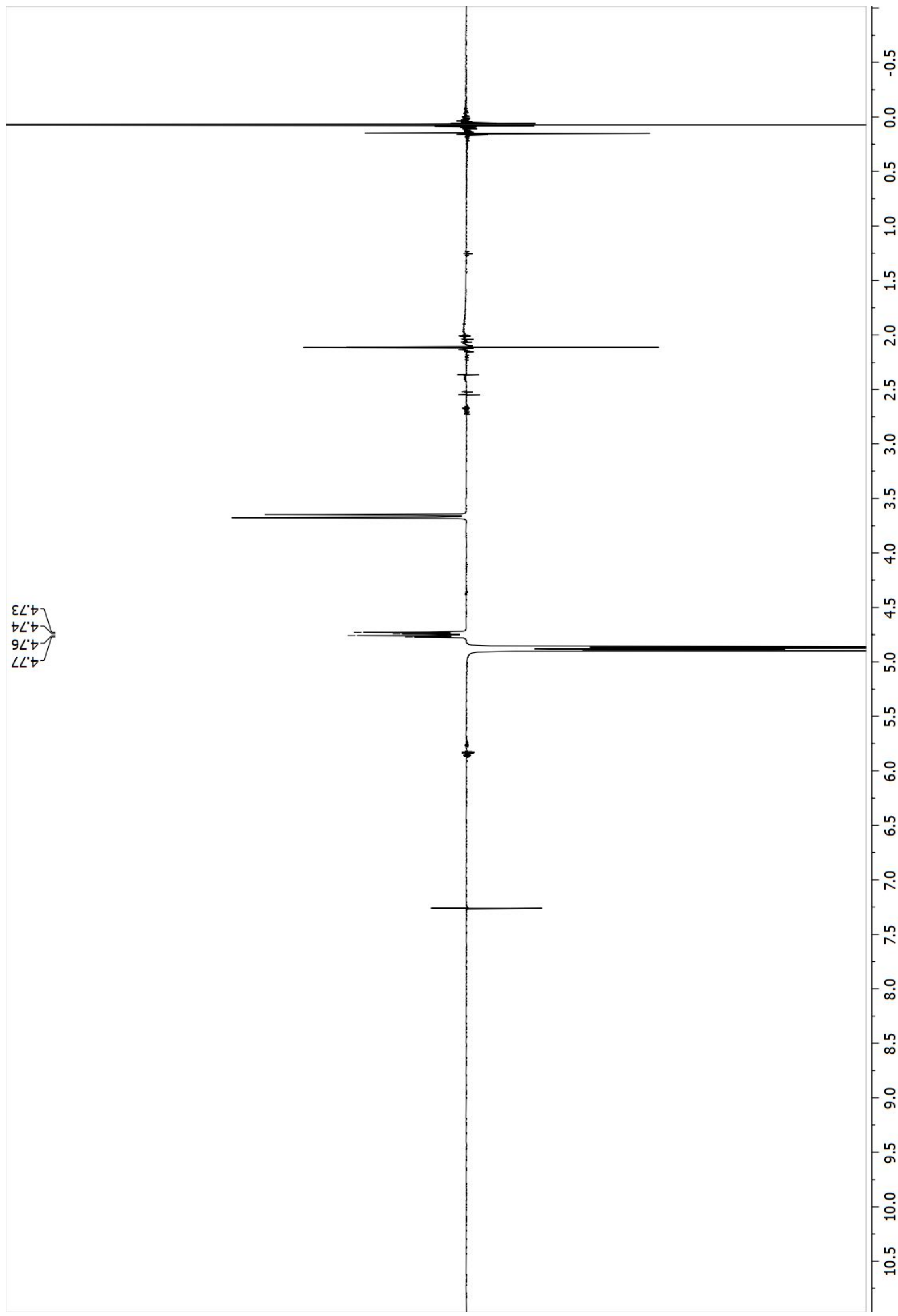

Spectrum 11 Nuclear Overhauser effects with excitation of the signal at 4.90 ppm measured in CDCl3 at 400 MHz.

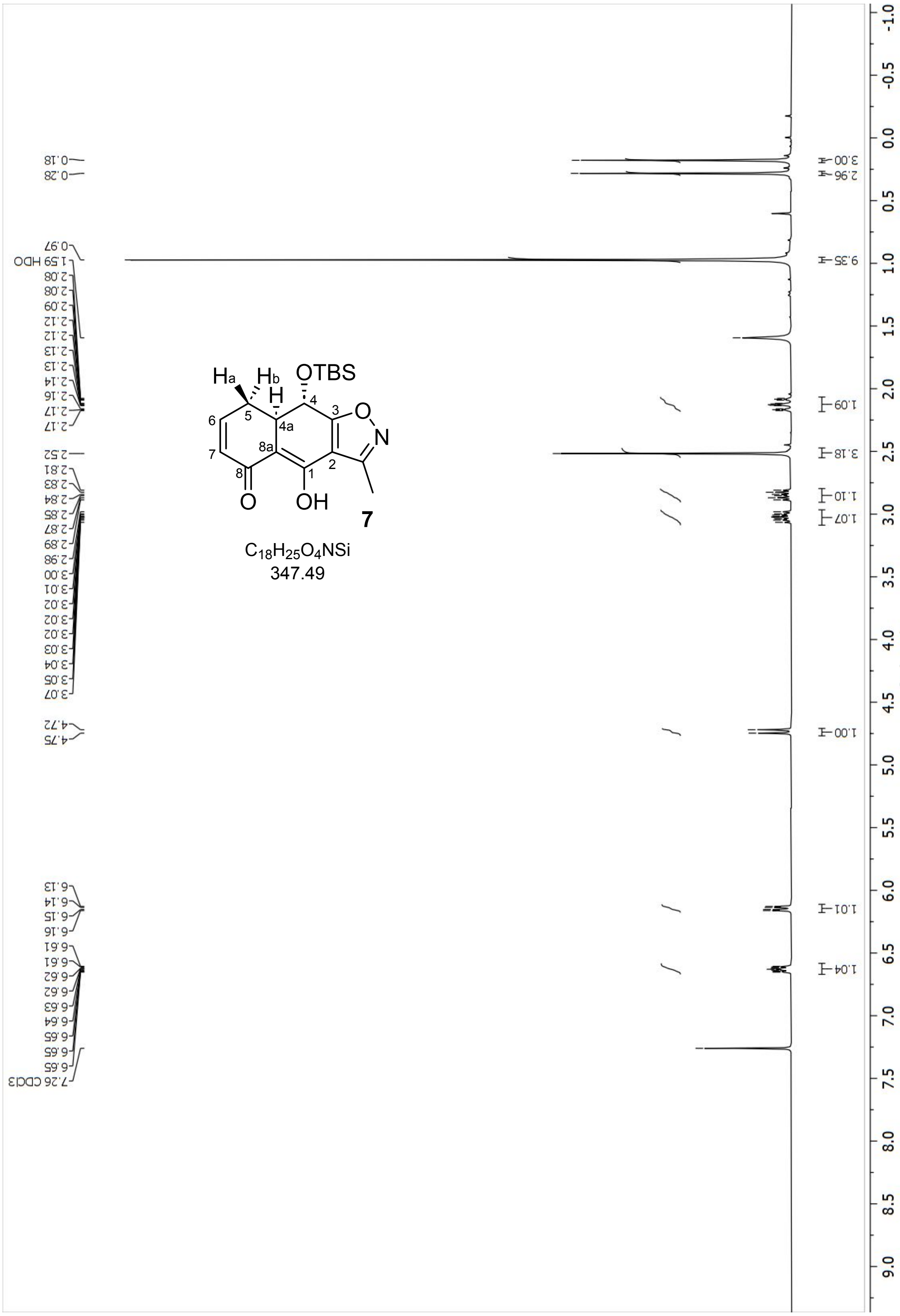

Spectrum 12 ¹H-NMR spectrum of compound **7** measured in $CDCl_3$ at 400 MHz.

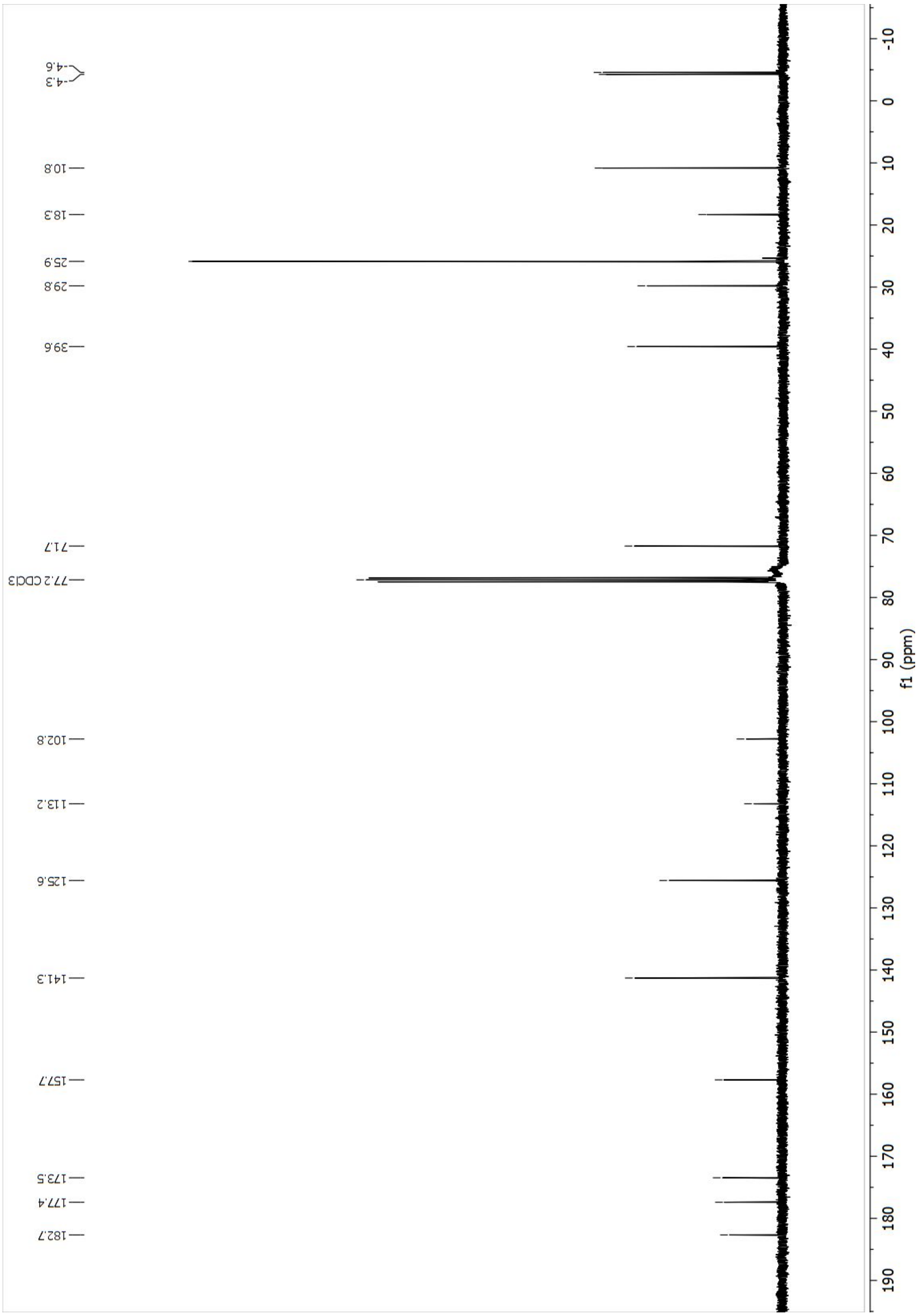

Spectrum 13 ^{13}C-NMR spectrum of compound **7** measured in $CDCl_3$ at 101 MHz.

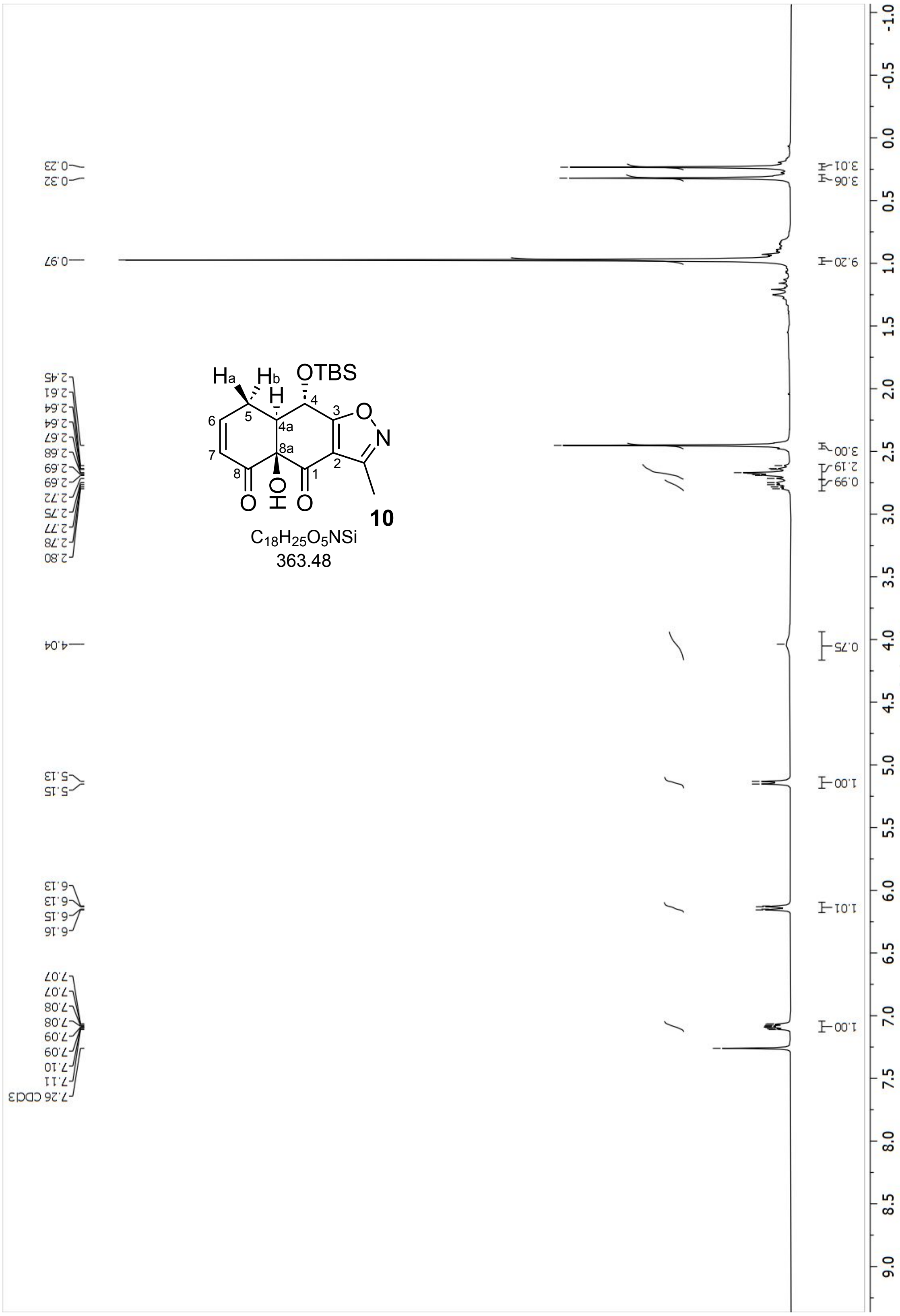

Spectrum 14 ^{1}H-NMR spectrum of compound **10** measured in $CDCl_3$ at 400 MHz.

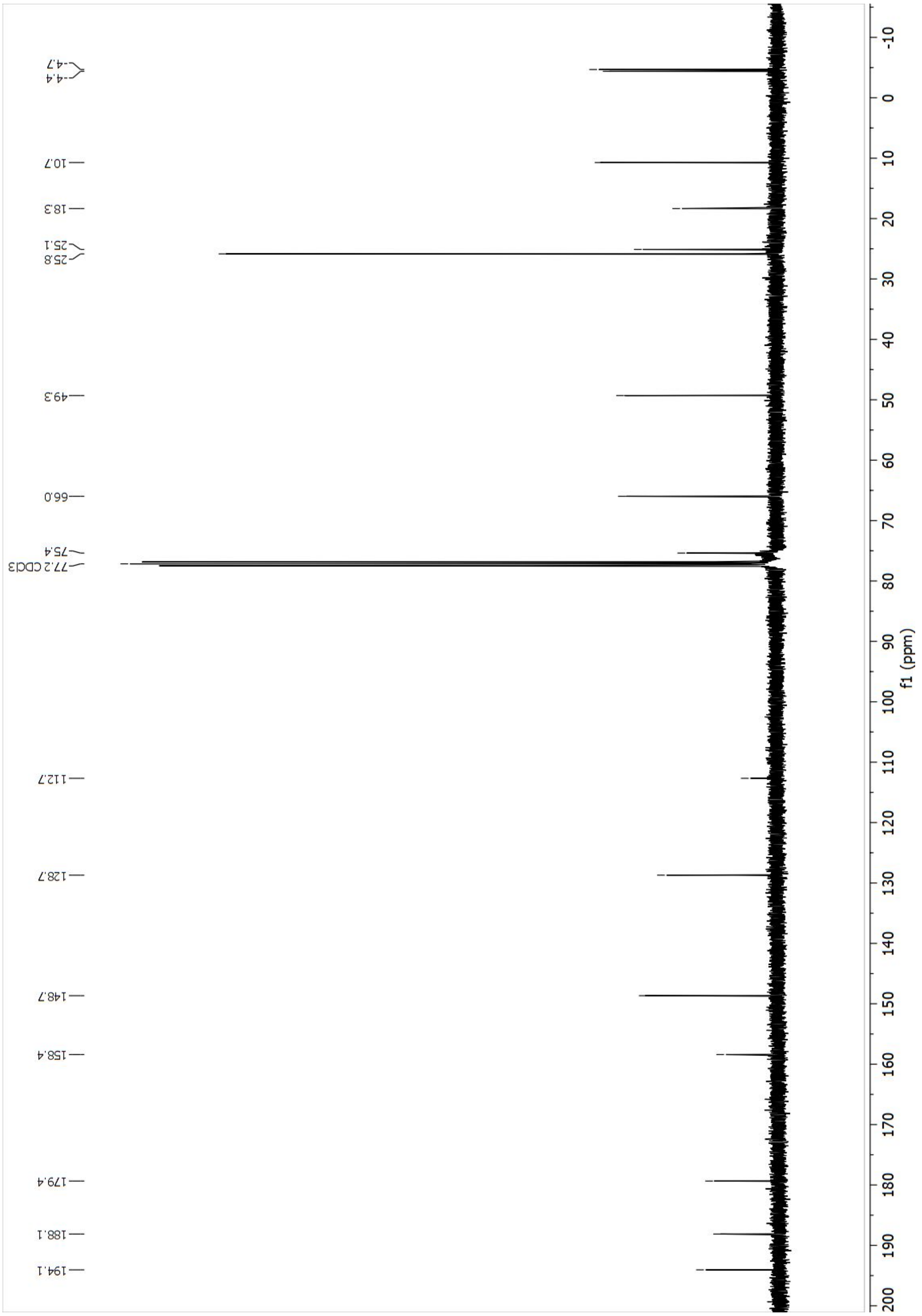

Spectrum 15 ^{13}C-NMR spectrum of compound **10** measured in $CDCl_3$ at 101 MHz.

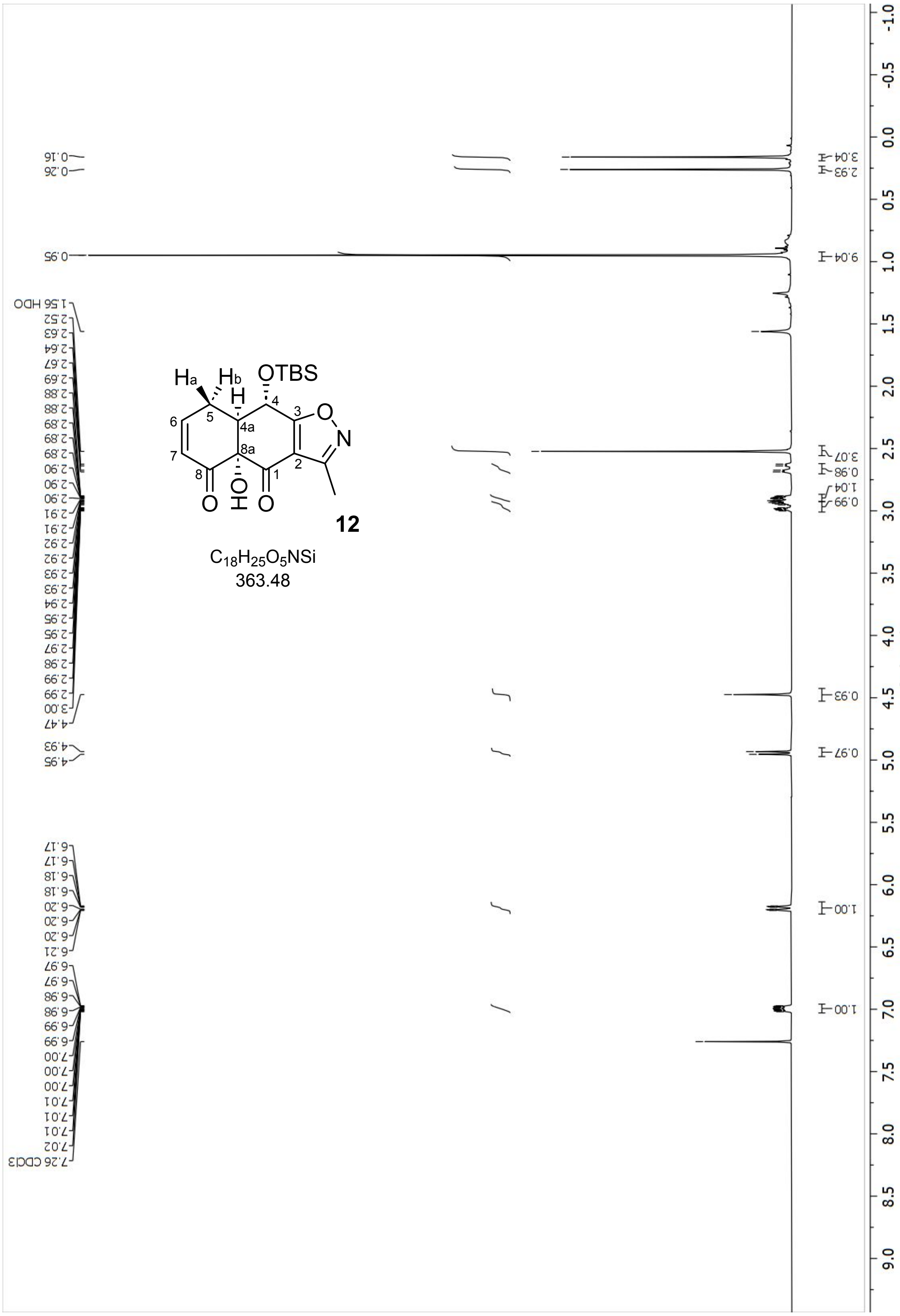

Spectrum 16 ^{1}H-NMR spectrum of compound **12** measured in $CDCl_3$ at 400 MHz.

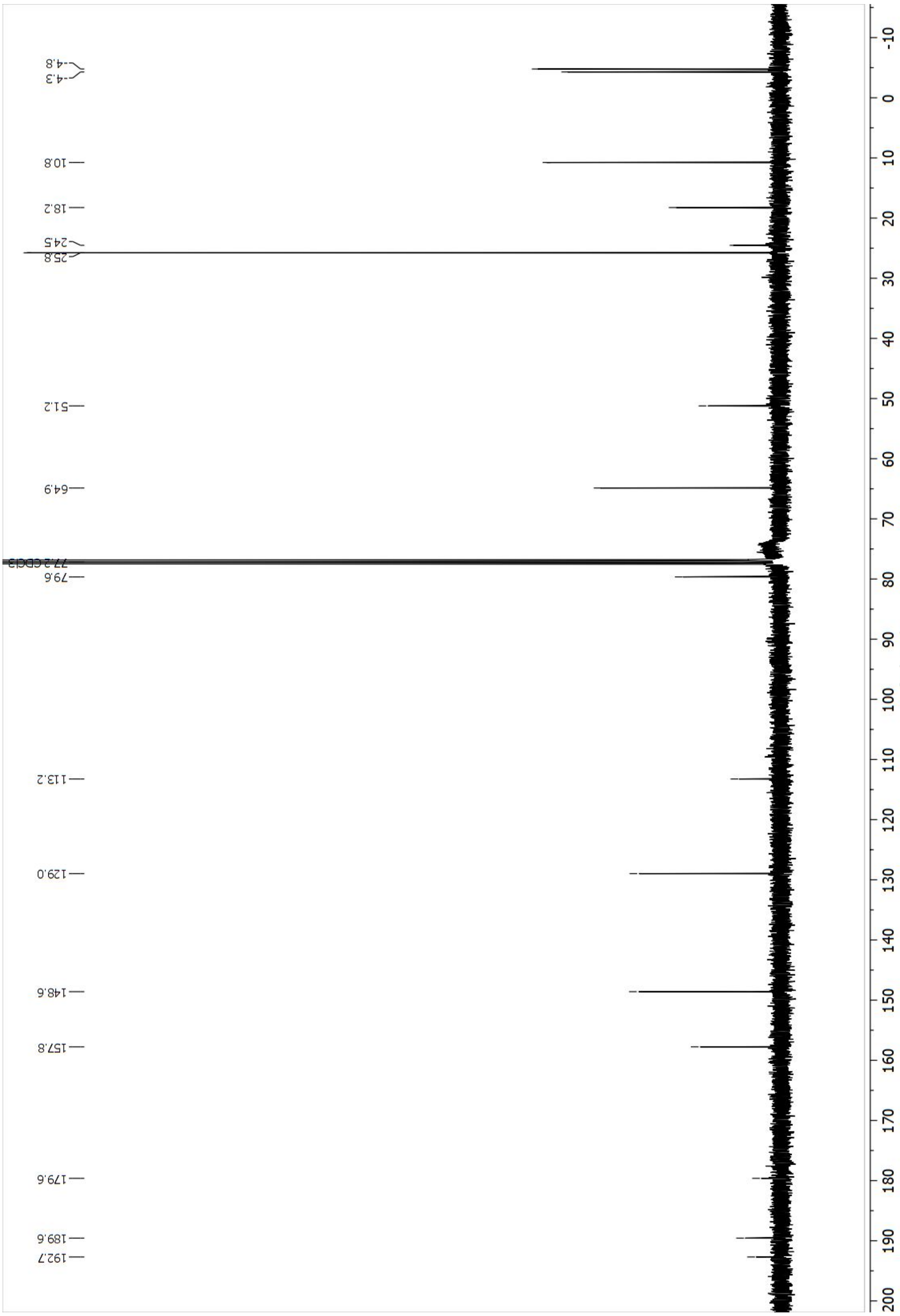

Spectrum 17 ^{13}C-NMR spectrum of compound **12** measured in $CDCl_3$ at 101 MHz.

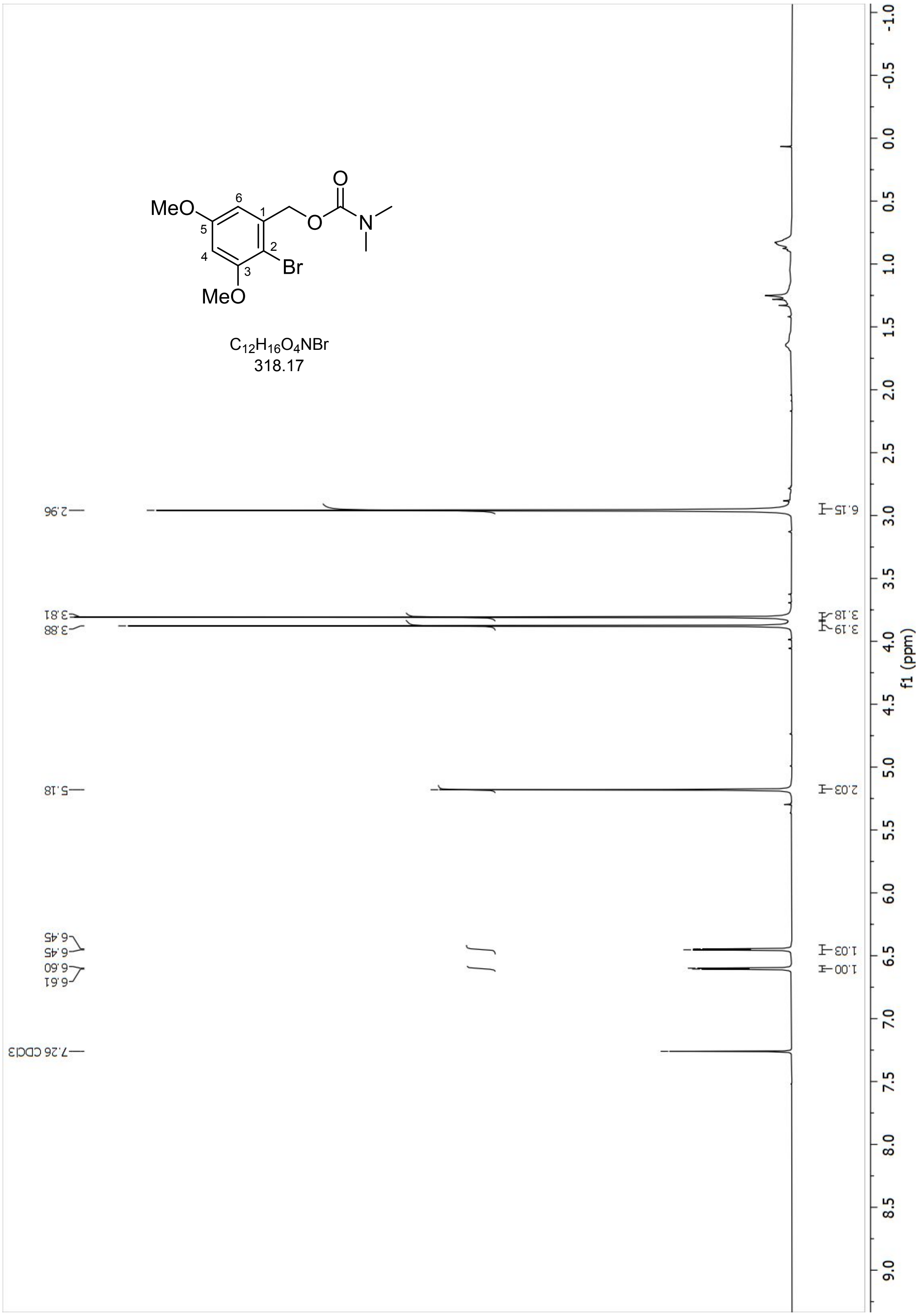

Spectrum 18 ^{1}H-NMR spectrum of the precursor to phthalide **17** measured in $CDCl_3$ at 400 MHz.

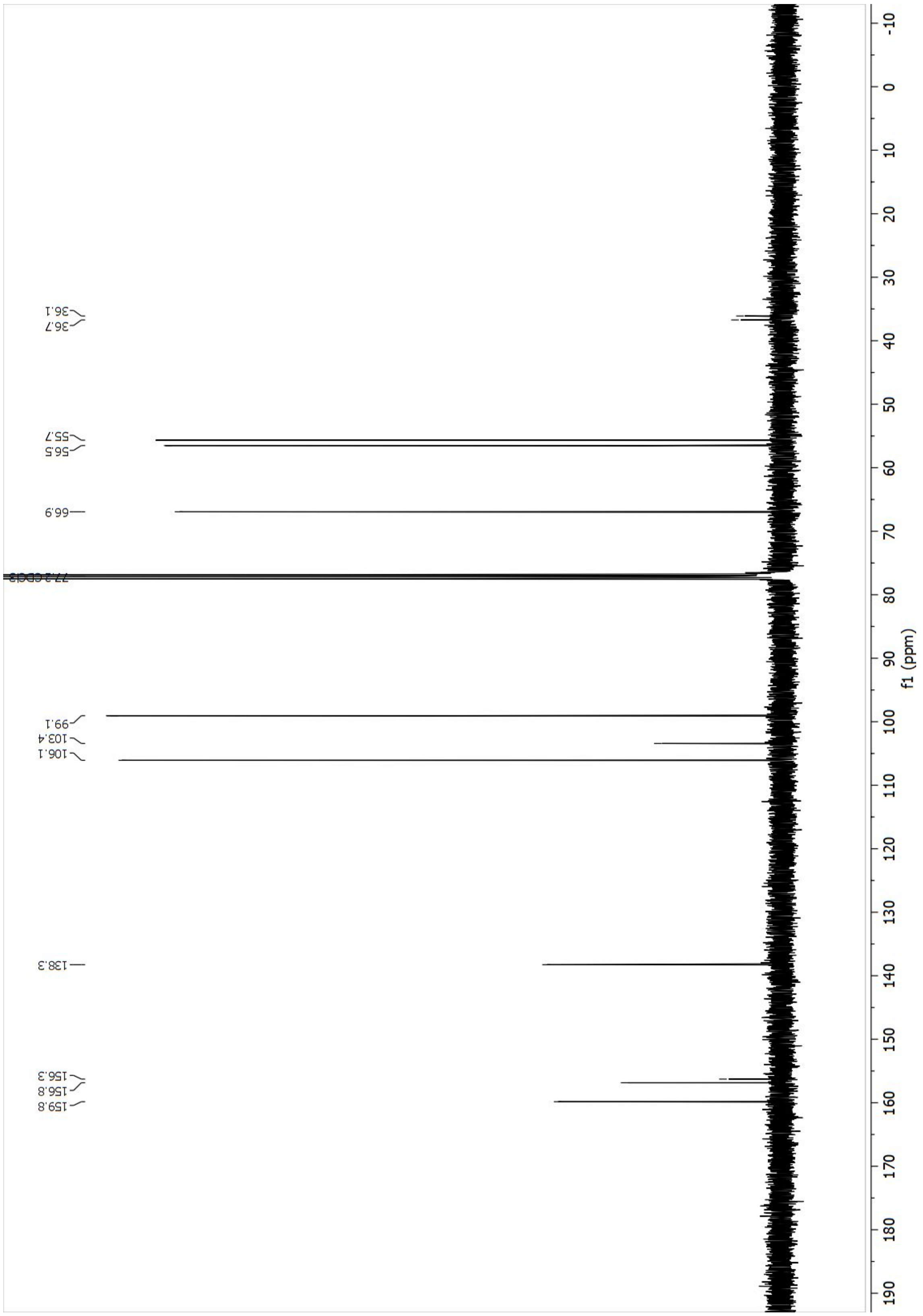

Spectrum 19 ^{13}C-NMR spectrum of the precursor to phthalide **17** measured in $CDCl_3$ at 101 MHz.

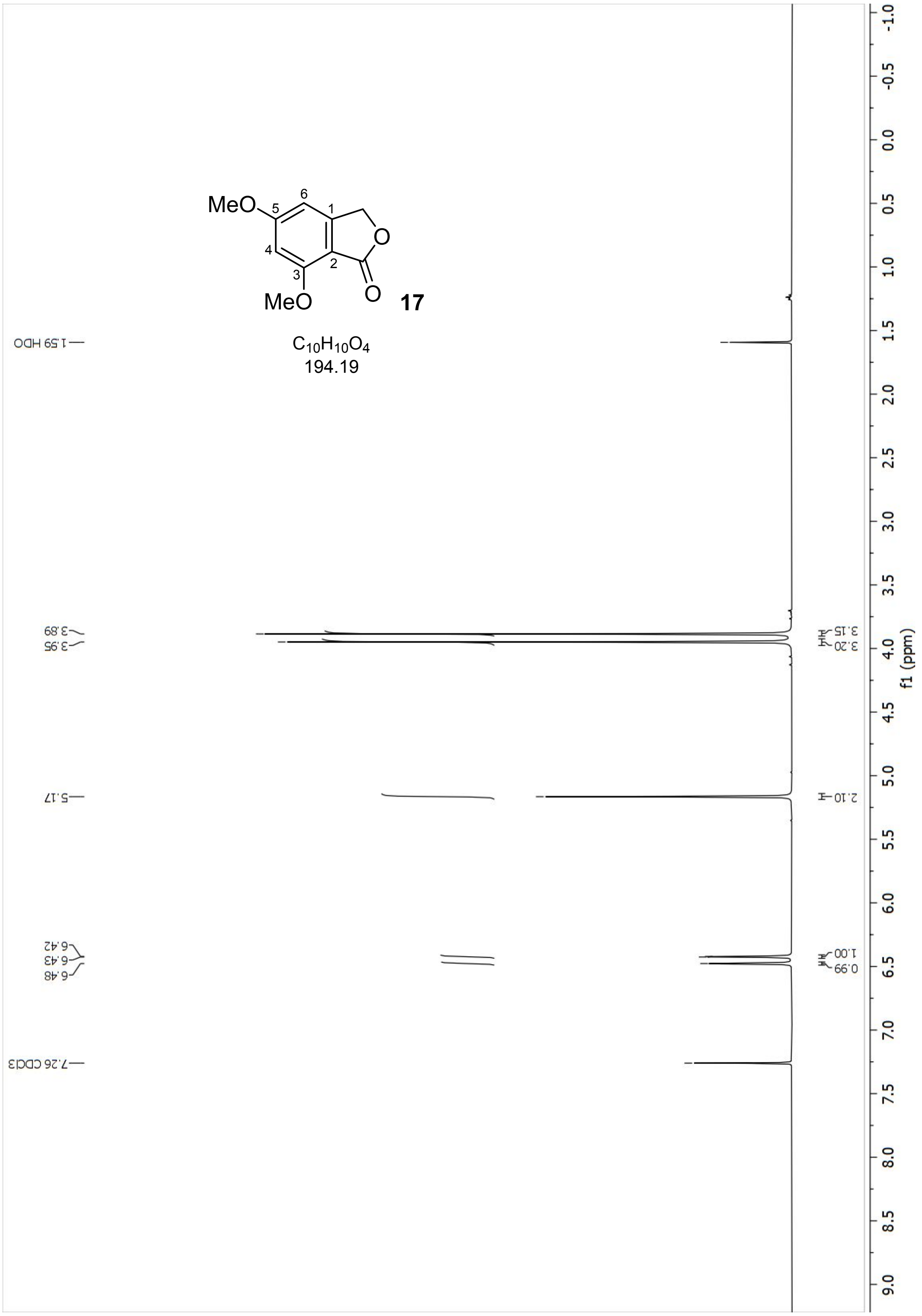

Spectrum 20 ^{1}H-NMR spectrum of compound **17** measured in $CDCl_3$ at 400 MHz.

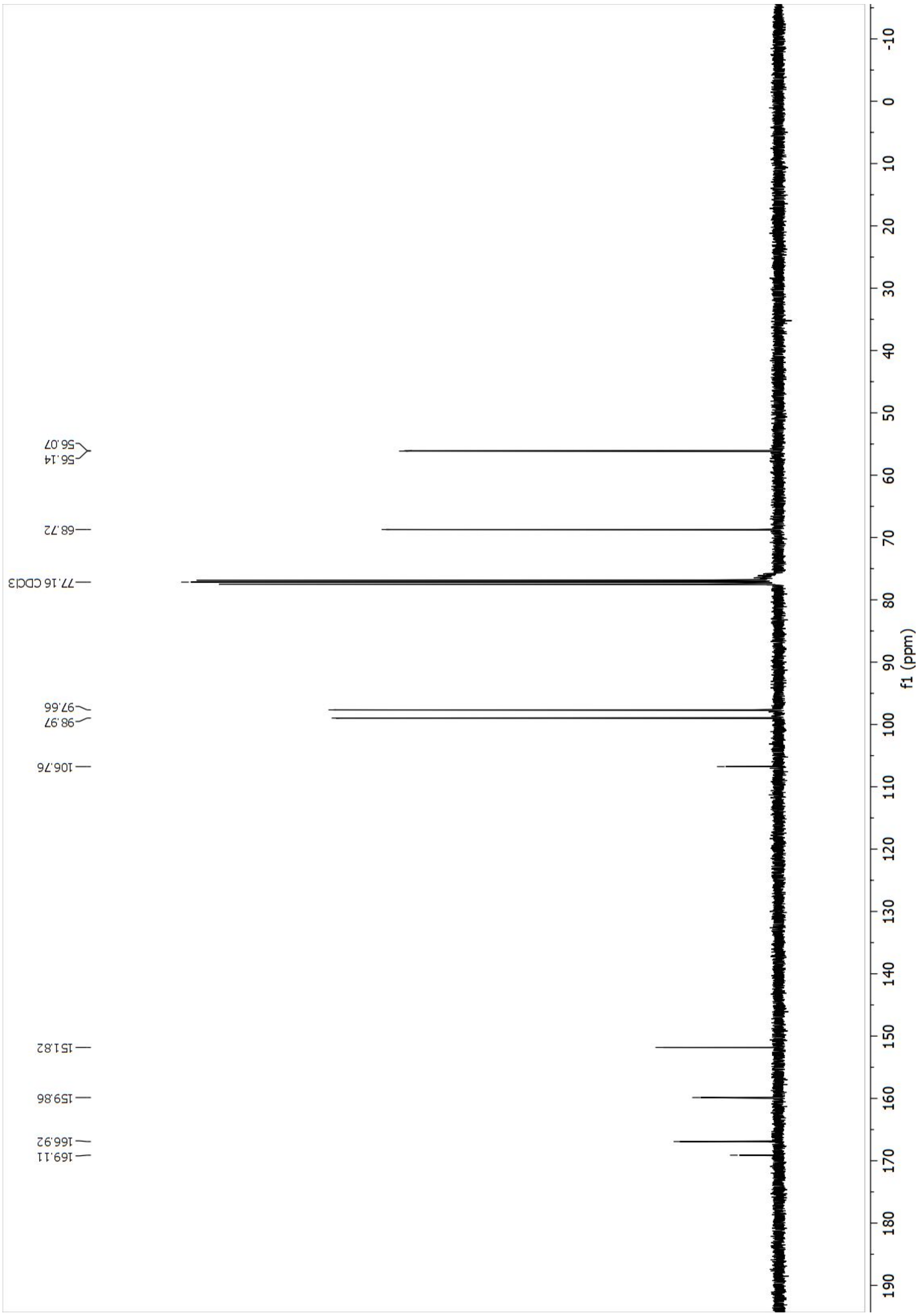

Spectrum 21 ^{13}C-NMR spectrum of compound **17** measured in $CDCl_3$ at 101 MHz.

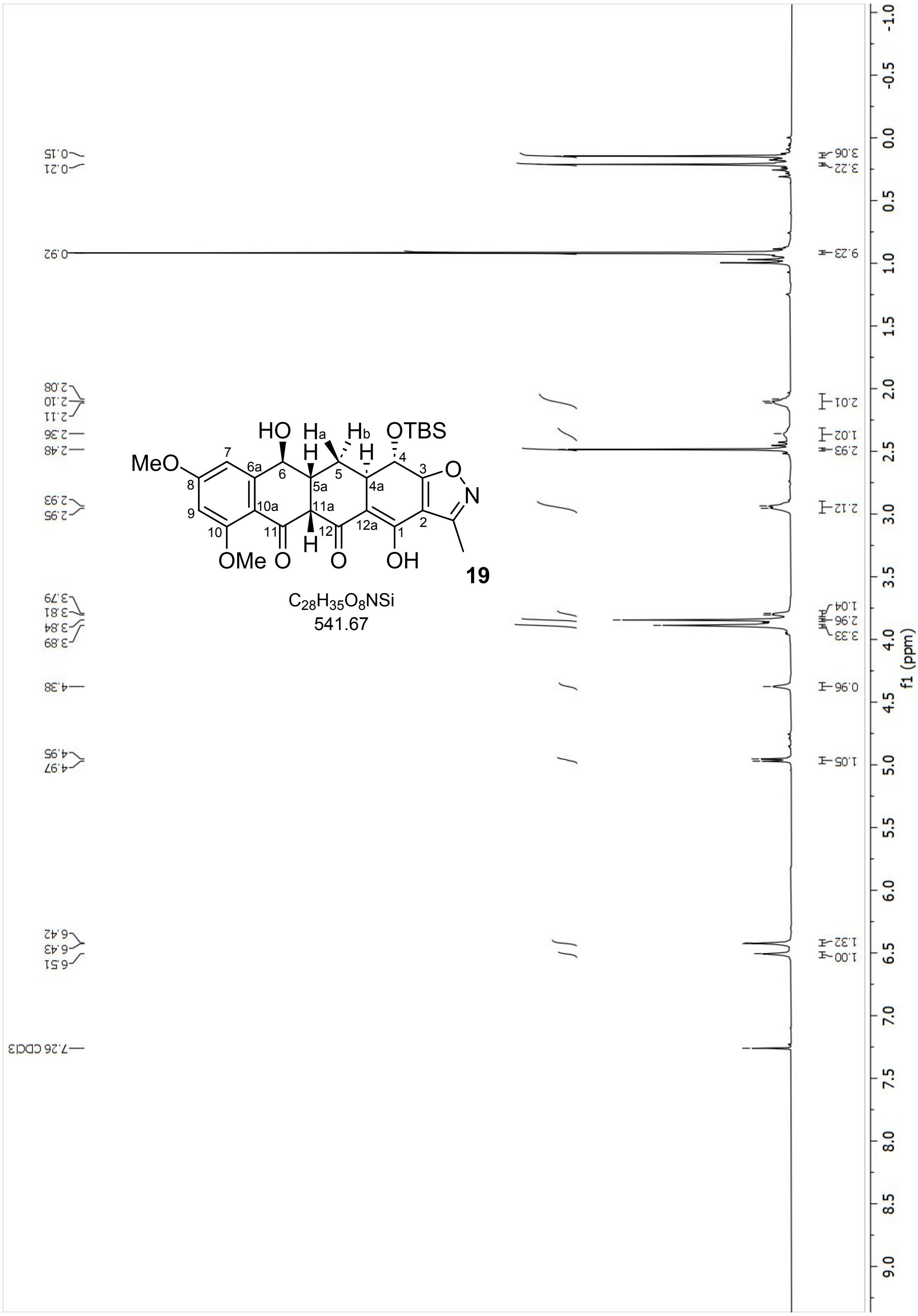

Spectrum 22 ^{1}H-NMR spectrum of compound **19** measured in $CDCl_3$ at 400 MHz.

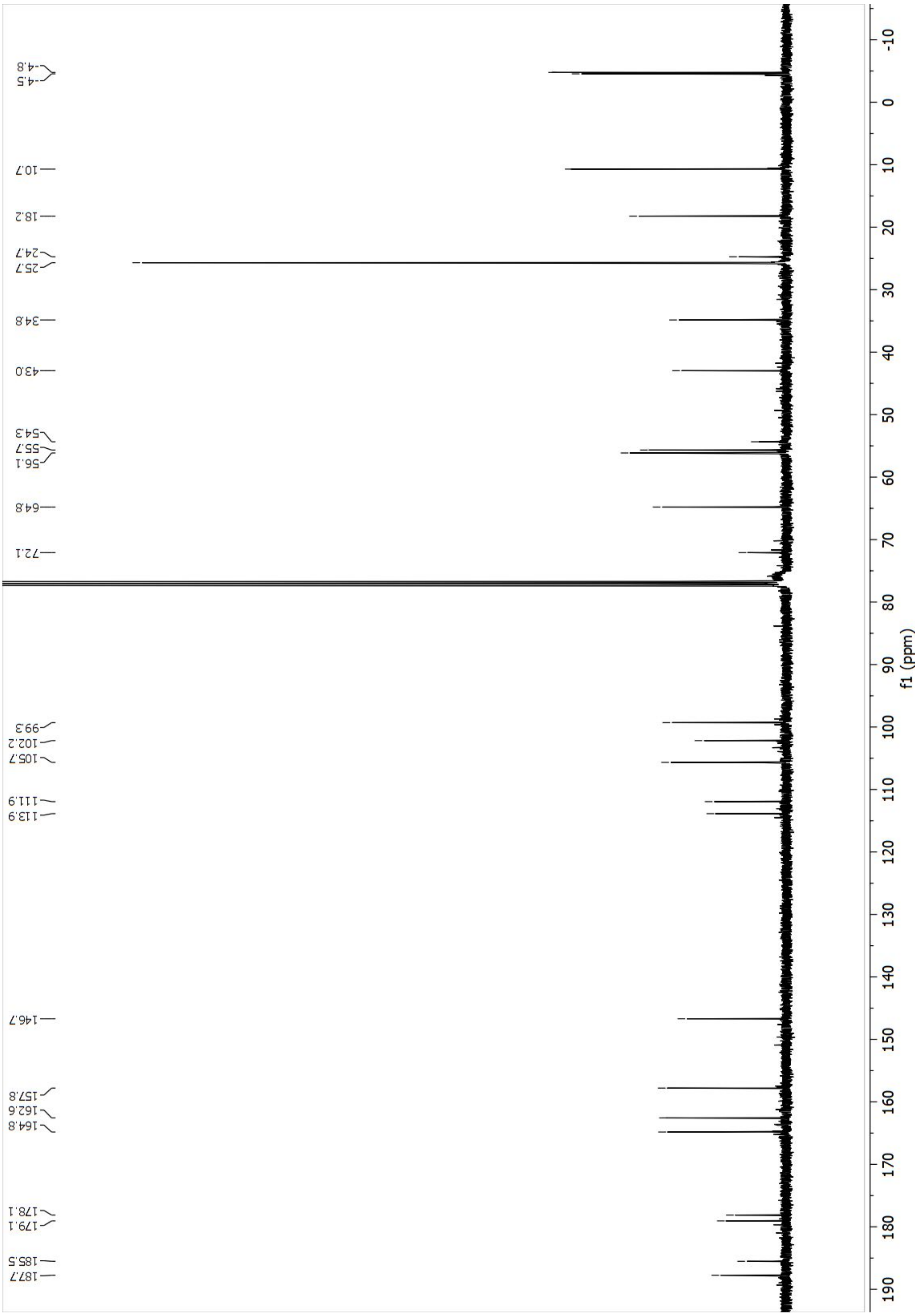

Spectrum 23 ^{13}C-NMR spectrum of compound **19** measured in $CDCl_3$ at 101 MHz.

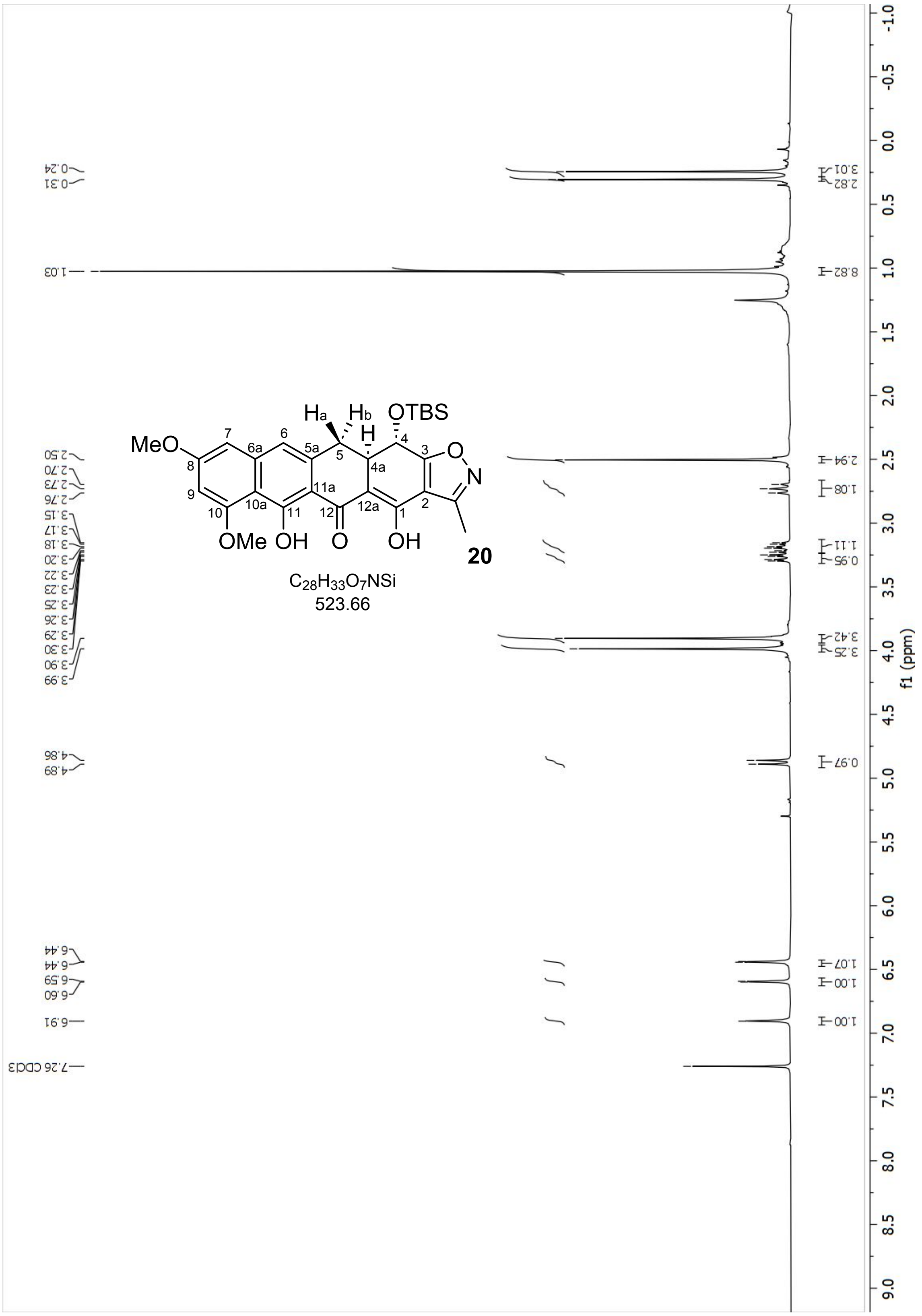

Spectrum 24 ^{1}H-NMR spectrum of compound **20** measured in $CDCl_3$ at 400 MHz.

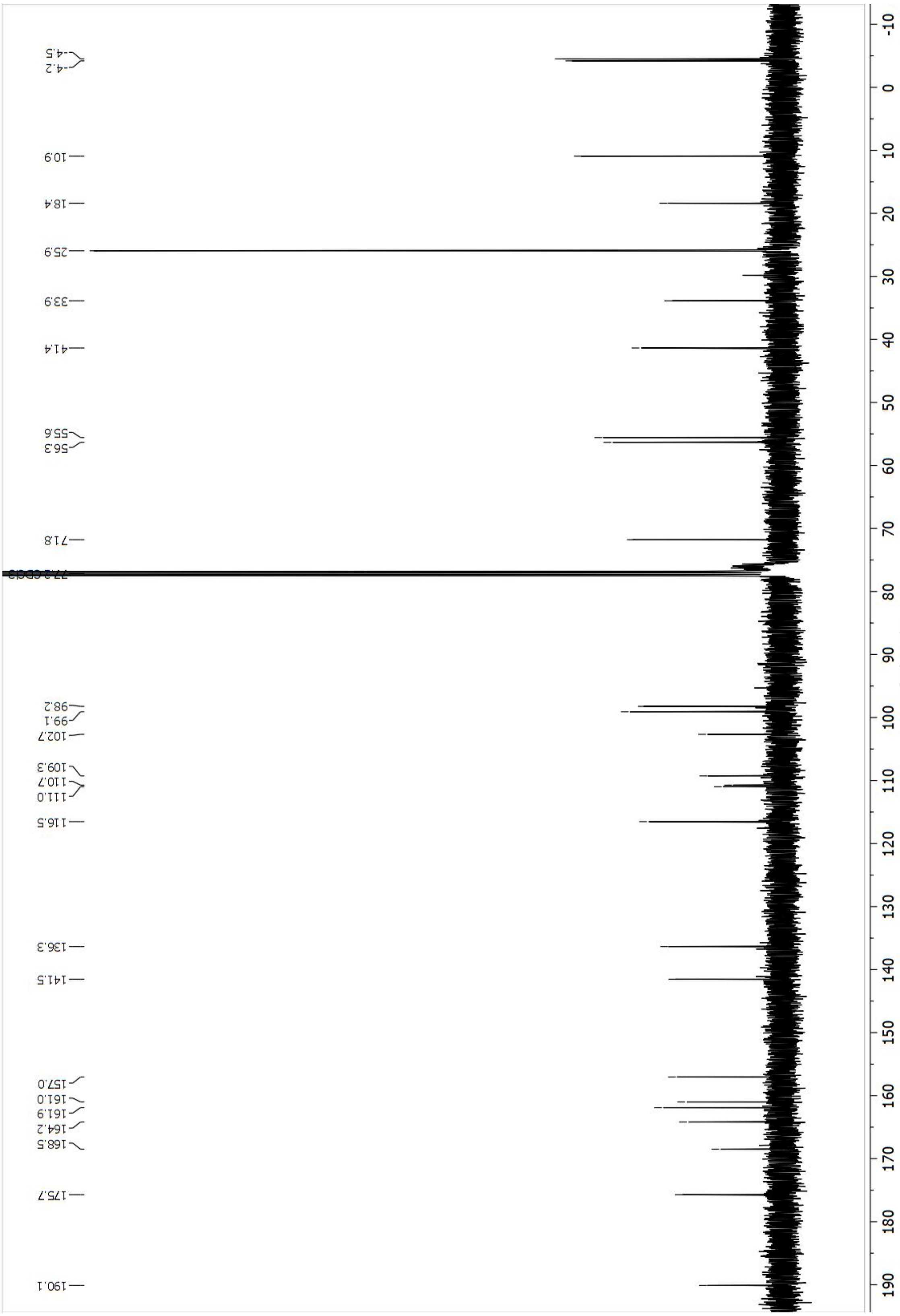

Spectrum 25 ^{13}C-NMR spectrum of compound **20** measured in $CDCl_3$ at 101 MHz.

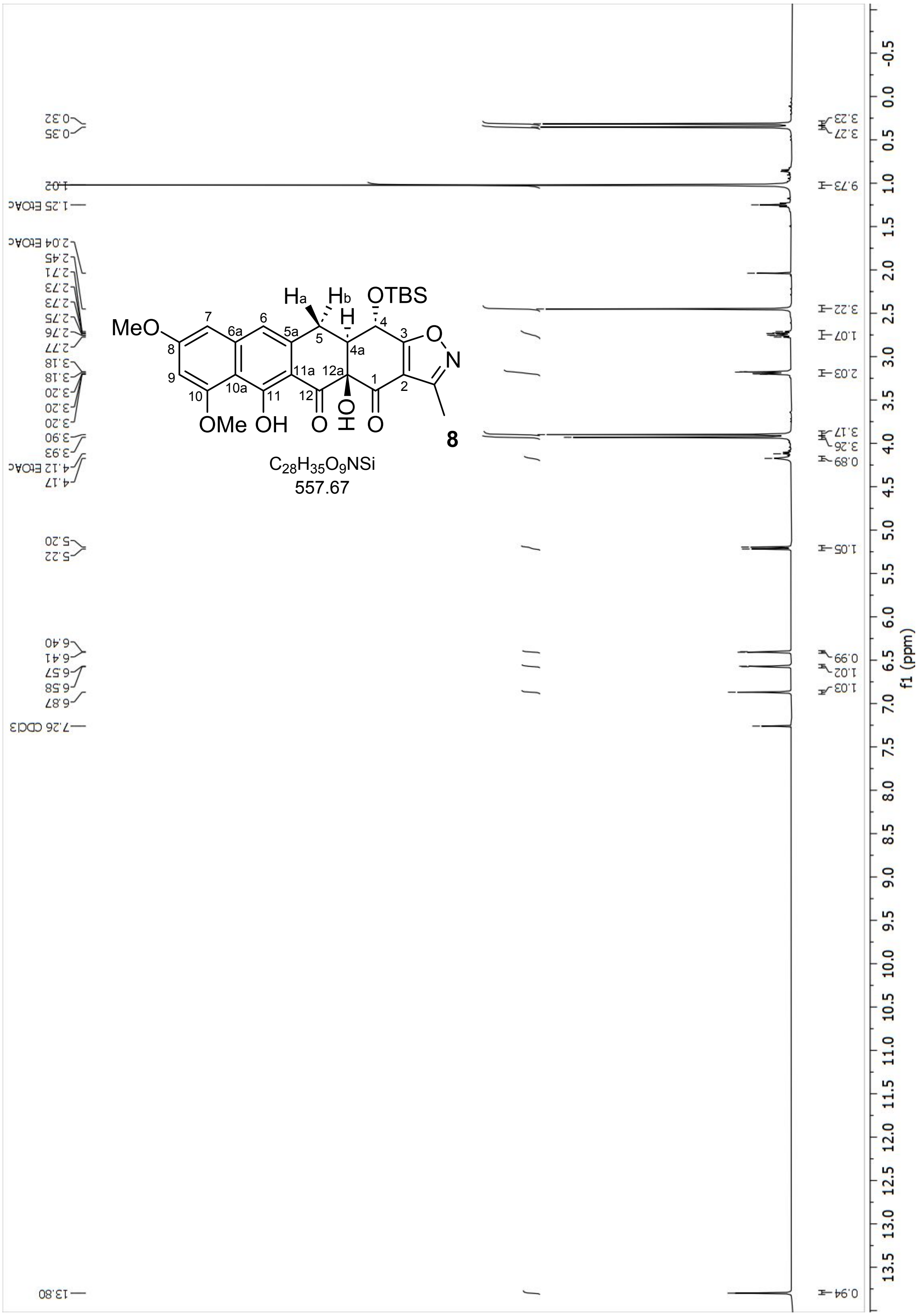

Spectrum 26 ^{1}H-NMR spectrum of compound **8** measured in $CDCl_3$ at 400 MHz.

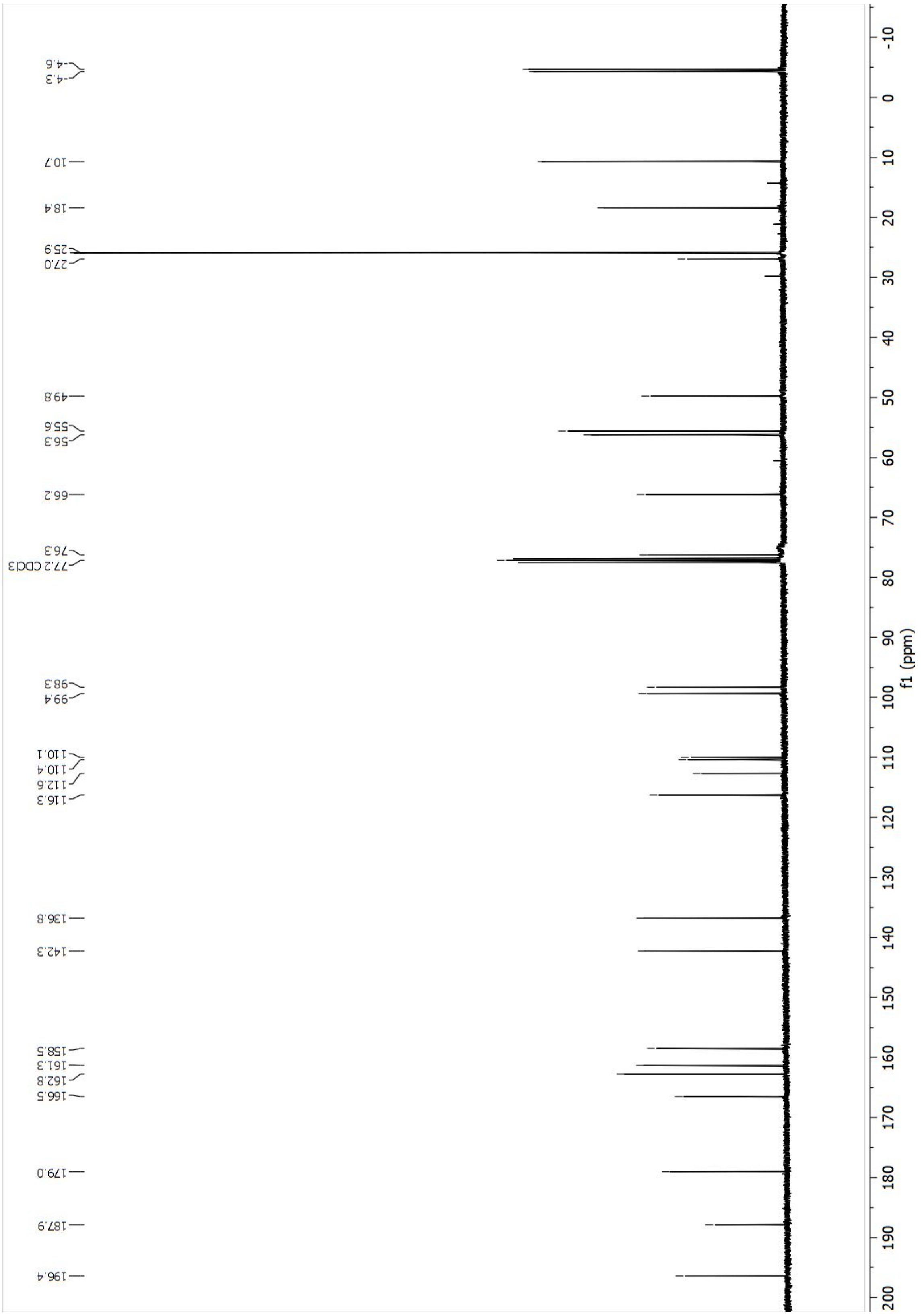

Spectrum 27 ^{13}C-NMR spectrum of compound **8** measured in $CDCl_3$ at 101 MHz.

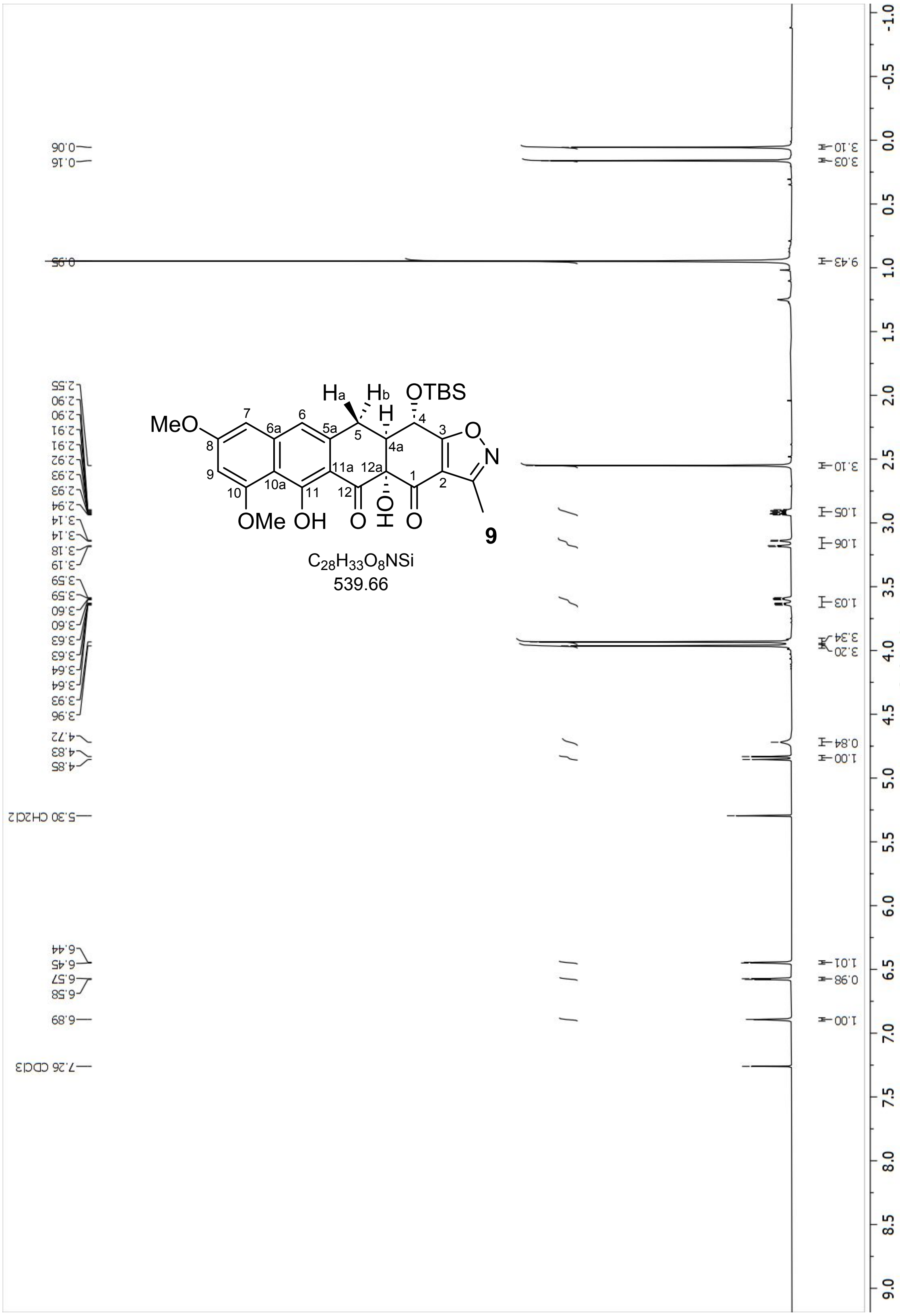

Spectrum 28 ^{1}H-NMR spectrum of compound **9** measured in $CDCl_3$ at 400 MHz.

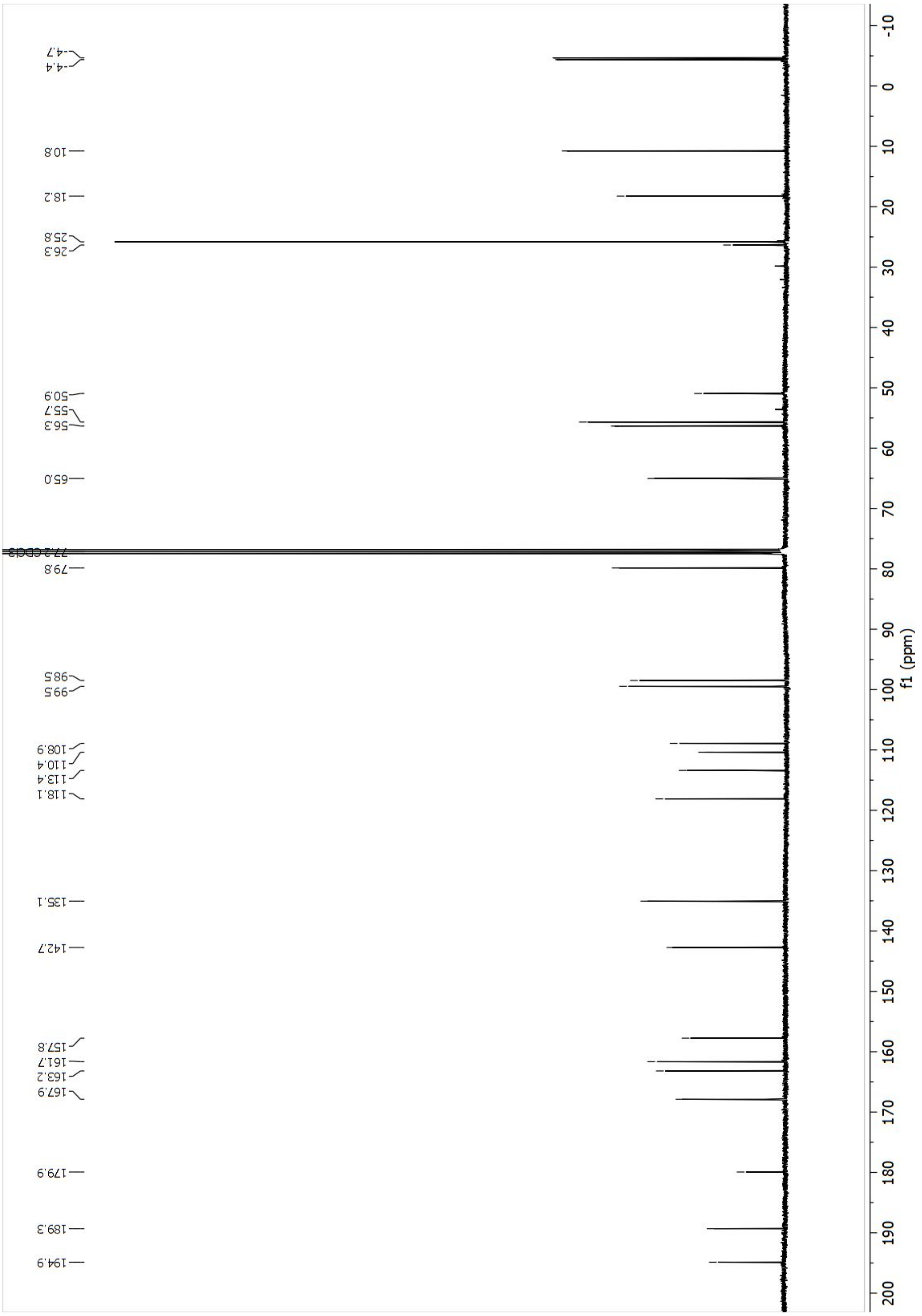

Spectrum 29 ^{13}C-NMR spectrum of compound **9** measured in $CDCl_3$ at 101 MHz.

4.2 Crystallographic parameters

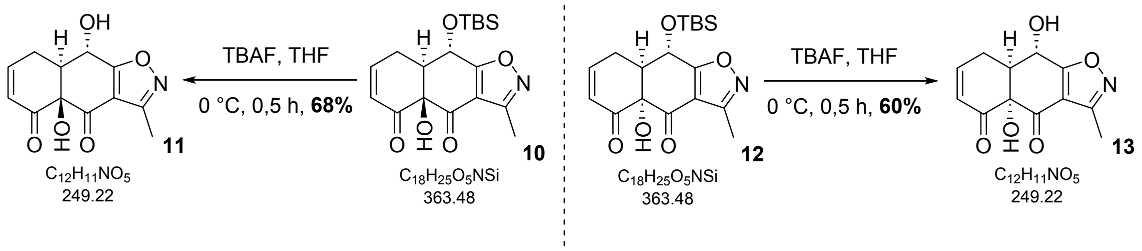

general method for TBS-group cleavage and crystallization (synthesis of diols **11** and **13**): Ketols **10** and **12** (40.0 mg, 0.11 mmol, 1.0 equiv.) were each dissolved in 1.1 ml THF and the solutions cooled to 0 °C. A 1M (THF) TBAF-solution (120 µl, 0.12 mmol, 1.1 equiv.) was then added and the solutions stirred for 30 minutes. The solutions were then allowed to warm to room temperature, diluted each with 5 ml of water and the products extracted with EtOAc (3x5 ml). The combined organic phases were dried over Na_2SO_4 and the solvent removed under reduced pressure affording diols **11** (18.6 mg, 0.08 mmol, **68%**; $\mathbf{R_f}$ = 0.22 (PE/ EtOAc = 3:2 (v/v))) and **13** (16.5 mg, 0.07 mmol, **60%**; $\mathbf{R_f}$ = 0.43 (PE/ EtOAc = 3:2 (v/v))). The crude compounds were each dissolved in 1.8 ml of a MTBE/ MeOH-mixture (1:1 (v/v)) and allowed to slowly crystalize by following the vapor diffusion technique (PE).

Crystal data and structure refinement for ketol **11**

Formula	$C_{12}H_{11}NO_5$
$D_{calc.}$/ g cm^{-3}	1.526
m/mm^{-1}	0.121
Formula Weight	249.22 g/mol
Color	colorless
Shape	needels
Size	0.180 x 0.400 x 0.820
T/K	200(2)
Crystal System	monoclinic
Flack Parameter	-0.3(6)
Space group	*P* 1 21 1
a/Å	6.548(5)
b/Å	8.659(5)
c/Å	9.629(7)
α/°	90
β/°	96.46(2)
γ/°	90
V/Å^3	542.5(6)
Z	2
Wavelength/Å	0.71073
Radiation type	Cu K_a
Θ_{min}/°	3.13
Θ_{max}/°	25.06
Measured Refl.	9924
Independent Refl.	1894
Reflections with I > 2σ(I)	1784
R_{int}	0.0585
Parameters	170
Restraints	1
Largest Peak/ e$^-$/Å^3	0.243
Deepest Hole/ e$^-$/Å^3	-0.253
GooF	1.062
wR_2 (all data)	0.0977
wR_2	0.0953
R_1 (all data)	0.0401
R_1	0.0371

Crystal data and structure refinement for ketol **13**

Formula	$C_{13}H_{15}NO_6$ ($C_{12}H_{11}NO_5$ + MeOH)
$D_{calc.}$/ g cm^{-3}	1.455
m/mm^{-1}	0.989
Formula Weight	281.26 g/mol
Color	colorless
Shape	plate
Size/mm^3	0.60×0.40×0.04
T/K	99.99(10)
Crystal System	monoclinic
Flack Parameter	0.05(10)
Space Group	$P2_1$
a/Å	5.9220(3)
b/Å	8.6852(3)
c/Å	12.4826(6)
α/°	90
β/°	91.361(4)
γ/°	90
V/Å^3	641.85(5)
Z	2
Wavelength/Å	1.54184
Radiation type	Cu K_a
Θ_{min}/°	3.542
Θ_{max}/°	78.817
Measured Refl.	13088
Independent Refl.	2727
Reflections with I > 2σ(I)	2663
R_{int}	0.0410
Parameters	203
Restraints	1
Largest Peak/ e$^-$/Å^3	0.363
Deepest Hole/ e$^-$/Å^3	-0.283
GooF	1.064
wR_2 (all data)	0.0903
wR_2	0.0894
R_1 (all data)	0.0347
R_1	0.0338

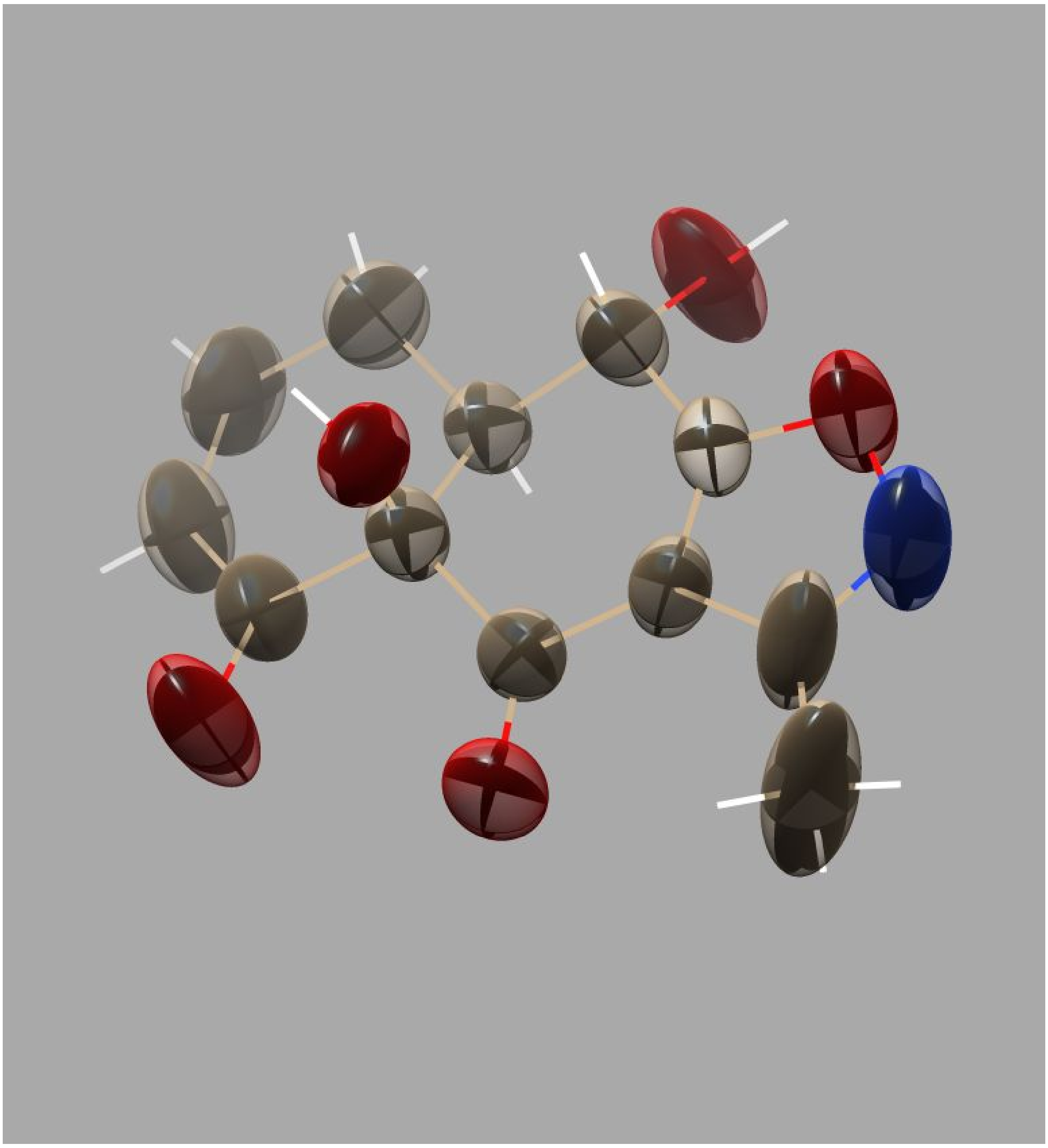

Fig. 1 Thermal ellipsoid plot of ketol **11** at the 30% probability level.

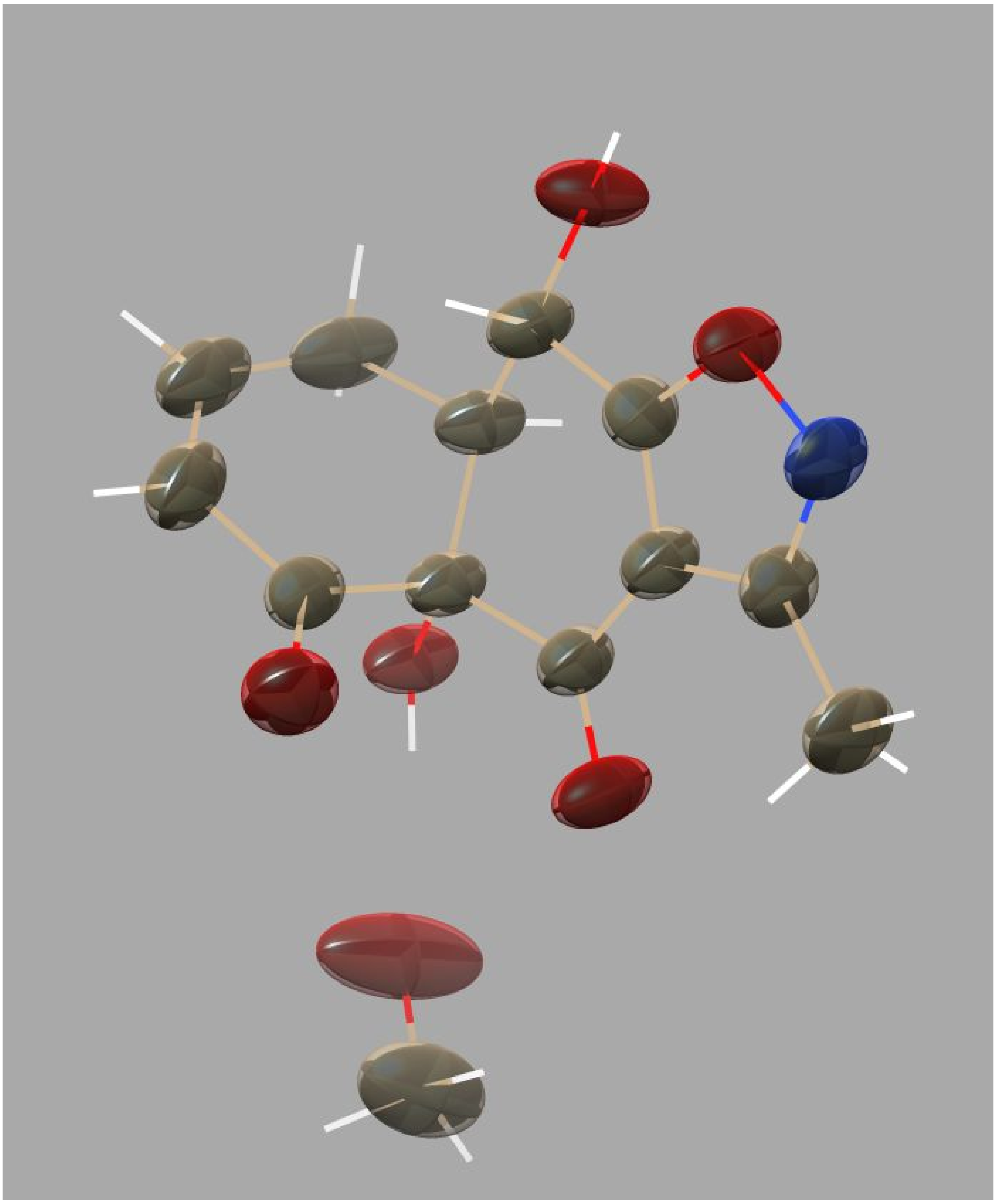

Fig. 2 Thermal ellipsoid plot of ketol **13** at the 30% probability level.
